# ﻿Diversity and distribution of the Trichoptera of Florida, United States, with descriptions of five new species

**DOI:** 10.3897/zookeys.1263.147317

**Published:** 2025-12-10

**Authors:** Andrew K. Rasmussen, Dana R. Denson, Alexander B. Orfinger, Steven C. Harris

**Affiliations:** 1 Center for Water Resources, Florida A&M University, Tallahassee, FL 32307, USA Center for Water Resources, Florida A&M University Tallahassee United States of America; 2 Central Florida Tourism Oversight District (retired), Lake Buena Vista, FL 32830, USA Central Florida Tourism Oversight District (retired) Lake Buena Vista United States of America; 3 Department of Life Science, Dalton State College, Dalton, GA 30720, USA Dalton State College Dalton United States of America; 4 Research Associate, Section of Invertebrate Zoology, Carnegie Museum of Natural History, Pittsburgh, PA 15213, USA Carnegie Museum of Natural History Pittsburgh United States of America

**Keywords:** Aquatic, biodiversity, caddisflies, conservation, faunistics, inventory

## Abstract

Based on previously published species accounts and collection records of the authors, a county-level distributional checklist of all 220 caddisfly species documented within Florida is presented, representing 46 genera within 19 families. New state records are provided for four species and five new Integripalpian species are described and illustrated: *Protoptila chipolensis* Rasmussen & Harris, **sp. nov.** (Glossosomatidae), *Hydroptila aviforma* Rasmussen & Harris, **sp. nov.** (Hydroptilidae), *Beraea jennyae* Rasmussen & Harris, **sp. nov.** (Beraeidae), *Ceraclea pescadori* Rasmussen & Harris, **sp. nov.** (Leptoceridae), and *Oecetis densoni* Rasmussen & Harris, **sp. nov.** (Leptoceridae). The most speciose families within Florida are the Hydroptilidae (77 species), Leptoceridae (54 species), Hydropsychidae (21 species) and Polycentropodidae (18 species). In addition to county-level distributional data, conservation status ranks and ranking recommendations for the newly described species are also provided. The majority of Florida’s caddisfly species are native to the eastern Nearctic, with many endemic (precinctive) to the Southeastern Coastal Plain, including 34 species known only from Florida. Diversity and distributional data are summarized for each family and regional diversity is compared between the panhandle and peninsula. Taxa richness and endemism are higher in the panhandle than in the peninsula. The panhandle contains 213 recorded species with 23 species endemic to the region, compared to the peninsula containing 131 recorded species, with five of those endemic to the peninsula. The higher taxonomic richness and presence of many cool-adapted taxa within the panhandle is likely due the temperate climate, diverse lentic and lotic habitats, and connectedness of river basins that extend north into Alabama and Georgia. The water bodies in counties of the Florida peninsula have a less diverse caddisfly fauna with taxonomic richness generally decreasing north to south along the peninsula, where habitat diversity declines and mean annual temperature increases.

## ﻿Introduction

The spatial distributions of the Earths’ organisms are often viewed in the context of geographic areas delimited by arbitrary political boundaries (e.g., nations, states/provinces, counties/parishes). Even though such areas are not natural regions per se, faunal surveys and inventories of them are useful for better understanding, managing, and conserving biotic diversity at various scales, including watershed, ecoregion, biomes, and biogeographic regions (e.g., Nearctic, Neotropical). With this in mind, we report new findings on the Trichoptera fauna of Florida and provide a summary and synthesis of the species diversity and distribution of species collected from counties within the state.

### ﻿Natural setting

The state of Florida, United States, is part of the Southeastern Coastal Plain of the North American continent and extends as a peninsula southward between the Gulf of Mexico and Atlantic Ocean from about 30°–25° North latitude. The northwestern portion of Florida, often referred to as the “panhandle”, is connected to northern areas of the Gulf Coastal Plain and upland physiographic provinces by river basins that drain large areas within the states of Alabama and Georgia to the North. These watershed connections are thought to have served as important corridors by which biota have moved north and south over millennia in response to climate changes during the Pleistocene and other epochs (Delcourt and Delcourt 1984). The movement of biota along mesic river corridors has resulted in geographic isolation of species within refugia and subsequent speciation, creating regions with high numbers of narrow-range endemic plants and animals that are recognized as biodiversity hot spots within Florida, especially the western Florida panhandle, which was ranked as one of the top five biodiversity hot spots of the continental United States (Chaplin et al. 2000; Rasmussen 2004). The diverse aquatic ecosystems found in Florida are tied to rich aquatic resources, including outstanding streams, rivers, springs, lakes, ponds, wetlands and large aquifers fed by 100–165 cm of rainfall per year (Fernald and Purdom 1998). Although Florida (maximum elevation of 105 m above sea level) is located on the lower Southeastern Coastal Plain, various geologic and topographic features (e.g., karst topography, river escarpments, sand hills and ridges) afford a wide variety of lotic and lentic habitats. Diverse climatic conditions also exist across the state, with the Florida panhandle being more temperate than tropical, as compared to the peninsula where the climate becomes more tropical in a north to south direction. The temperate and subtropical climates associated with the Florida panhandle and peninsula, respectively, create different thermal regimes. Water bodies of the Florida panhandle experience much cooler ambient temperatures in the winter than do water bodies on the peninsula where winter temperatures and average yearly temperatures increase along a north to south temperature gradient.

Trichoptera (caddisflies) are an order of holometabolous insects that live primarily in aquatic habitats during their immature life stages, after which the adults emerge and mate terrestrially within riparian areas. With more than 16,000 described extant species, Trichoptera have more species than the other primary aquatic insect orders (Ephemeroptera, Plecoptera, Odonata) combined. On the North American continent 1511 extant species, representing 27 families and 156 genera, have been reported from the United States and Canada (Rasmussen and Morse 2023). Trichoptera in Florida have been the subject of investigation by entomologists beginning in the mid-1800s with *Banksiola concatenata* (Phryganeidae) reported from St. John’s Bluff, East Florida by Walker (1852) and *Nectopsyche candida* (Leptoceridae) described by Hagen (1861) being the first Florida species with the primary type collected within the state. Since that time, trichopterologists have reported and described species from the state in numerous publications. Of special note, beginning in the 1980s co-author Steven C. Harris and his colleagues have described 39 new species of Trichoptera that occur in the state, including 28 species of microcaddisflies (Hydroptilidae). For a comprehensive list of the publications documenting each species within the state, we refer readers to the ‘Distributional Checklist of Nearctic Trichoptera’ which is periodically updated and available online at https://trichoptera.org/. As with many insect groups, alpha taxonomy of Trichoptera is based primarily on adult morphology, with a heavy emphasis placed on features of the male genitalia. Larvae have been associated and described for only approximately 50% of the Trichoptera species of Florida (Pescador et al. 2004).

### ﻿Field surveys and checklists

Field surveys of Trichoptera in Florida have been conducted by several investigators beginning in the 1960s, including: a survey of Hydroptilidae at 23 localities (Blickle 1962); insects, including Trichoptera, of the Archbold Biological Station (Frost 1966); Trichoptera of three streams on Eglin Air Force Base (Harris et al. 1982); Trichoptera of North Florida ravine streams (Rasmussen 2004); a statewide survey of Trichoptera species, with a focus on species of special conservation need (Rasmussen et al. 2008a); and Trichoptera of the Chipola River basin (Denson et al. 2016). Field surveys and species inventories by AKR, DRD, and colleagues primarily employed UV-blacklight pan traps to capture adult specimens. The first preliminary checklist of Florida Trichoptera listed 120 species within the state (Gordon 1984) and was presented in 1983 at the 4^th^ International Symposium on Trichoptera held in Clemson, South Carolina. Additional checklists of Florida species were presented in larval identification manuals by Pescador et al. (1995, 2004) with 175 and 192 Florida species listed, respectively. More recently, Harris et al. (2012) presented an annotated checklist (76 spp.) of the Hydroptilidae of Florida, with descriptions of five new species.

### ﻿Conservation status assessments

The Florida Natural Areas Inventory (**FNAI**), founded in 1991, is a member of The Nature Conservancy’s international network of natural heritage programs, now coordinated by NatureServe. FNAI scientists currently collect information and “track” 50 species of Florida Trichoptera and provide state-level conservation status assessments following NatureServe protocols. Prior to FNAI, The Florida Committee on Rare and Endangered Plants and Animals (**FCREPA**) produced a series of publications titled Rare and Endangered Biota of Florida, including two volumes on the invertebrates that included conservation assessments of Florida Trichoptera species prepared by John C. Morse (Morse 1982, 1994). The species assessed in those reports formed the basis of the species tracked by FNAI. In addition to the work of FNAI, the Florida Fish and Wildlife Conservation Commission has developed a list of Species of Greatest Conservation Need (**SGCN**) as part of the State Wildlife Action Plan. Rasmussen et al. (2008a) addressed considerable information gaps and provided conservation status assessments of 29 species of Trichoptera designated as SGCN species at the time. More recently, Rasmussen and Harris (2016) provided conservation status assessments of five species of Trichoptera as part of a review by the U.S. Fish and Wildlife Service to determine if federal listing of these species is warranted under the U.S. Endangered Species Act.

Despite considerable efforts by biologists to document the Trichoptera of Florida, our comprehension of Trichoptera biodiversity in the state remains fragmented and with a rudimentary research focus on documenting what species occur in Florida and where. We seek here to augment this knowledge base by presenting new findings and synthesizing current knowledge on the species composition, conservation status, and distribution of the Florida fauna. Our hope is that this work will serve to facilitate future research on these fascinating creatures and is useful in terms of bettering the management and protection of aquatic habitats and the species they support.

## ﻿Materials and methods

### ﻿Species descriptions

Specimens were preserved in 80% ethanol and examined using Olympus SZX16 and Leitz Wild M3Z stereomicroscopes. To better observe internal and external structures of the male and female genitalia, abdomens were removed and cleared of soft tissue by placement in a 10% KOH solution. Pencil sketches were produced from genitalia placed in a water soluble CMCP 9/9F mounting medium and viewed at 250X through a Leitz Laborlux S compound microscope; phalli were drawn separately and dissected out in some specimens. Additional details were added based on photographs of the genitalia and viewing the specimens under stereomicroscopes. Pencil sketches were scanned and used as base templates to produce the final illustrations in Adobe Illustrator following the methods of Holzenthal (2010). Morphological terminology used for the genitalia of: *Protoptila chipolensis* sp. nov. follows that of Morse (1988) and Robertson and Holzenthal (2013); *Hydroptila aviforma* sp. nov. follows that of Marshall (1979); *Beraea jennyae* sp. nov. follows that of Schmid (1998); *Ceraclea pescadori* sp. nov. follows that of Morse (1975), Yang and Morse (1988), and Carnagey and Morse (2006); *Oecetis densoni* sp. nov. follows that of Chen (1993). Total specimen length for *P. chipolensis* sp. nov., *H. aviforma* sp. nov., and *B. jennyae* sp. nov. was measured from anterior margin of the head to the apex of the folded wings and reported as a range, mean, and number of specimens measured. Forewing length for *C. pescadori* sp. nov. and *O. densoni* sp. nov. was measured from the base to the apex and reported as a range, mean, and number of specimens measured. The materials and methods used in DNA barcoding and analysis of *Oecetis densoni* sp. nov. are presented along with the species description in the results section. Types are deposited, as indicated in the species descriptions in the following institutions:

**CMNH** Carnegie Museum of Natural History, Pittsburg, Pennsylvania

**CUAC** Clemson University Arthropod Collection, Clemson, South Carolina

**FAMU** Florida A&M University Aquatic Insect Collection

**NMNH** National Museum of Natural History, Smithsonian Institution, Washington, DC

**UMSP** University of Minnesota Insect Collection, St. Paul, Minnesota

### ﻿Checklist of Florida Trichoptera

**Figure 1.**
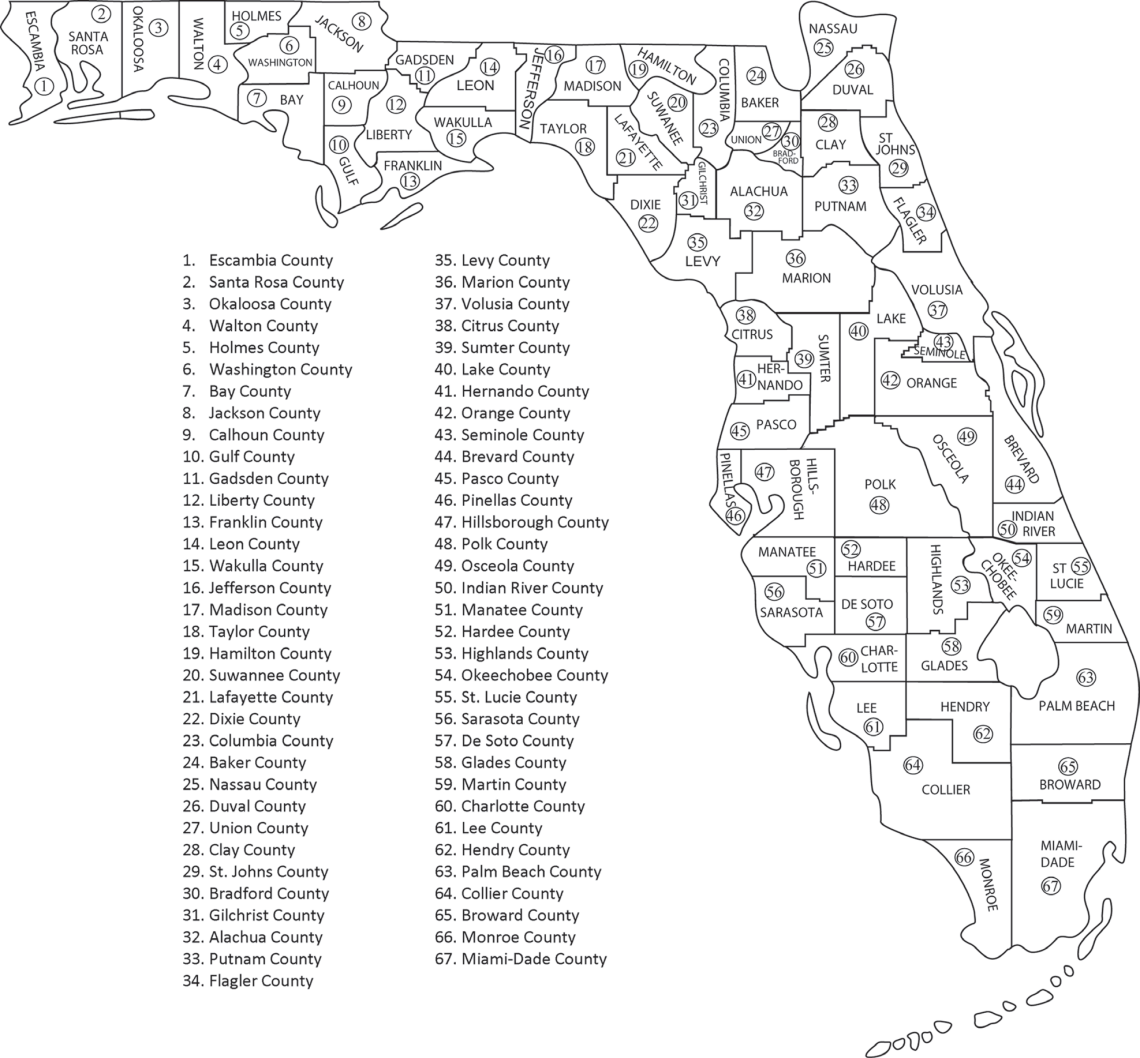
Counties of Florida with numbering corresponding to that used in Tables 1–3.

Species records from all published sources listed by Rasmussen and Morse (2023) and unpublished specimen records of AKR, DRD, and SCH were searched to determine the counties from which each species was collected. The 67 Florida counties were assigned numbers sequentially from east to west and north to south across the state (Fig. 1) following the system used by Harris et al. (2012). For purposes of this study, county #1–18 comprise the Florida “panhandle” and the remaining counties (#19–67) comprise the peninsula. The checklist of Florida Trichoptera species is presented in table format, with the species split into three tables, namely: Table 1Annulipalpia (fixed-retreat makers), Table 2Integripalpia (basal lineages), and Table 3Integripalpia (portable tube-case makers). Published reports of species from Florida that we believe are erroneous, or otherwise dubious, were identified and placed in Table 4. Within Tables 1–3, the species are grouped alphabetically by family and genus. Species recorded for the first time in Florida are preceded with an asterisk (*); species known only from the state of Florida are preceded with a house symbol (⌂); and species highlighted in bold font have their primary type specimens coming from Florida. In addition to the county records, Tables 1–3 also list the conservation status rankings for each species as assigned by NatureServe (2024). Additionally, conservation status rankings were counted and tabulated (Table 5). Authorship of species discussed below is provided either when the species is first mentioned or in Tables 1–3.

## ﻿Results and discussion

**Figure 2.**
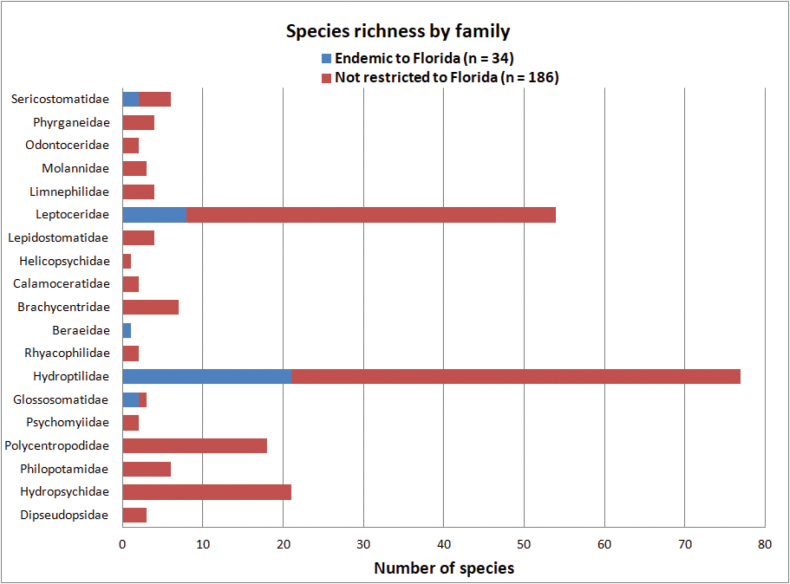
Species richness showing number of Florida endemic and non-endemic species within each family.

In total, we report 220 distinct species of Trichoptera from Florida placed within 46 genera and 19 families. The 220 species comprise 213 nominal species as well as seven species new to science. Thirty-four of the species we report are currently unknown from outside Florida and considered to be endemic (precinctive) to the state (Tables 1–3). The most species rich families (Fig. 2) are the Hydroptilidae (77 spp.), Leptoceridae (54 spp.), Hydropsychidae (21 spp.), and Polycentropodidae (18 spp.).

**Figure 3.**
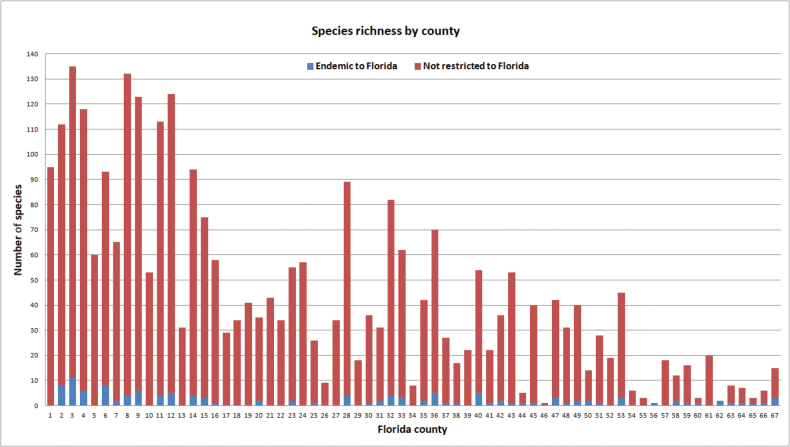
Species richness showing number of Florida endemic and non-endemic species within each of Florida’s 67 counties. County names are presented in Fig. 1.

Species richness for Florida counties ranged from one species to 135 species (Fig. 3). The wide range and large variation in county species richness (mean = 43.8, st. dev. = 36.5) is in part due to unequal sampling effort among counties. Nevertheless, the overall higher species richness recorded in panhandle counties and decline of species richness southward on the peninsula are underpinned by regional differences in faunal diversity. The total species richness for the Florida panhandle (counties 1–18) is 213 species of the 220 species recorded in the state, and is much higher than the total species richness for the peninsula (counties 19–67) where 131 species are reported. As noted in the family accounts below, many Florida species (*n* = 89) are restricted in Florida to the panhandle as compared far fewer species (*n* = 7) being restricted to the peninsula. The high diversity and uniqueness of the panhandle is also supported by the fact that 23 of 34 species of Florida endemics are restricted to the panhandle, as compared to five Florida endemics being restricted to the peninsula. The differences in alpha diversity between the two regions is reflected in the number of species recorded from panhandle counties (Fig. 3), which averaged 86 species per county as compared to an average of 28 species recorded from peninsula counties. Another pattern observed from county species richness data illustrated in Fig. 3 is that species richness is higher in northern counties of the peninsula as compared to southern counties. The species pool of the southern half of the state is comprised of warm adapted species within only five families and 19 genera as compared to the northern half of the state where species within 19 families and 46 genera are represented.

Below we provide original descriptions for five of the seven undescribed species. Next, an annotated checklist of the Trichoptera of Florida, organized by suborder and family, is presented (Tables 1–3, Suppl. material 1). After that, erroneous historical records of Florida Trichoptera are listed and annotated (Table 4). Lastly, the conservation status rankings (Table 5) are summarized.

### ﻿New species descriptions

#### ﻿Integripalpia: Glossosomatidae

***Protoptila* Banks**

##### ﻿ Protoptila chipolensis

Taxon classificationAnimaliaTrichopteraGlossosomatidae

Rasmussen & Harris sp. nov.

7BF5FD7A-0FD5-5291-A096-A29CA9461062

https://zoobank.org/A5C6BEA5-7E21-44B6-8398-CF3C567A1BF1

Fig. 4

Protoptila sp. nov.; collection records, Denson et al. (2016).

###### Type material.

***Holotype male*** (CMNH) • **Florida, Jackson County**, Chipola River at Caverns Road (State Road 166), 30°39'43"N, 85°13'21"W, 6 May 2010, D. Denson, E. Denson, UV pan trap. ***Paratypes*** • Same data as holotype, 20 males and 10 females (CMNH) • 20 males and 10 Females (CUAC) • 20 males and 10 females (FAMU) • 20 males and 10 females (NMNH) • 20 males and 10 females (UMSP); **Florida, Jackson County** • Rocky Creek at Highway 71, 18 May 1994, M. Pescador, S. Harris, 3 males and 1 female (FAMU) • Chipola River at State Road 167 near Marianna, 30°47'36"N, 85°13'18"W, 4 October 2006, D. Denson, UV pan trap, 10 males (CMNH) • Chipola River at State Road 162, 30°52'12"N, 85°15'32"W, 2 October 2010, D. Denson, E. Denson, UV pan trap, 14 males and 18 females (CUAC) • same as previous, except 6 August 2011, D. Denson, 19 males and 22 females (UMSP) • Chipola River at Peacock Bridge ramp, 30°37'36"N, 85°09'56"W, 21 May 2010, D. Denson, A. Rasmussen, UV pan trap, 25 males and 44 females (FAMU) • Pelt Creek at State Road 73, 30°39'43"N, 85°13'21"W, D. Denson, UV pan trap, 12 males and 20 females (NMNH) • Hollis Branch upstream of Chipola River, 30°33'13"N, 85°10'14"W, 21 May 2010, D. Denson, A. Rasmussen, UV pan trap, 8 males, 13 females (UMSP) • Bridge Creek at State Road 71, 30°39'05"N, 85°09'45"W, 7 May 2011, D. Denson, UV pan trap, 1 male (FAMU) • Waddell’s Mill Creek upstream of Chipola River, 30°51'03"N, 85°16'44"W, D. Denson, E. Denson, UV pan trap, 18 males and 108 females (CMNH); **Calhoun County** • Chipola River at Highway 20, 27 September 1972, P. Carlson 2 males 3 females (CUAC) • Chipola River at Highway 274, 30°32'03"N, 85°09'54"W, 17 May 1994, M. Pescador, S. Harris, R. Flowers, UV pan trap, 78 males and 27 females (FAMU) • Chipola River at boat ramp 5 km SW of Altha, 30°33'05"N, 85°10'17"W, 28 March 1998, M. Pescador, A. Rasmussen, UV pan trap, 4 males and 1 female (FAMU) • Chipola River at Laramore Landing, 30°30'44"N, 85°09'31"W, 20 May 2010, A. Rasmussen, D. Denson, D. Ray, R. Abad, L. Brooks, Mercury vapor sheet, 29 males and 12 females (UMSP) • same as previous, except 6 May 2011, D. Denson, UV pan trap, 7 males and 137 females (CUAC) • Chipola River at Look and Tremble Rapids, 30°31'29"N, 85°09'42"W, 6 May 2011, D. Denson, UV pan trap, 4 males and 37 females (CMNM) • same as previous, except 28 October 2011, D. Denson, E. Denson, 9 males and 22 females (FAMU).

###### Other material examined.

**Florida, Calhoun County** • Chipola River at boat ramp 5 km SW of Altha, 30°33'05"N, 85°10'17"W, 26 March 2010, A. Rasmussen, D. Denson, dipnet, 1 larva (FAMU) • Chipola River at Highway 274, 30°32'03"N, 85°09'54"W, 3 September 2010, A. Rasmussen, C. Zhou, dipnet, 3 larvae and 1 pupa (FAMU).

###### Diagnosis.

The male of *Protoptila chipolensis* sp. nov. is most similar to *P. maculata* (Hagen) in the narrow, elongate similarly-shaped sternum VIII and the thick phallic parameres that are sharply turned apically; the parameres of *P. chipolensis* sp. nov., however, are less thick than those of *P. maculata*. The new species is most easily distinguished from all other Nearctic *Protoptila* by the shape of Segment X, the arms of which are stout and strongly decurved in lateral view and terminate in a distinctive cuspidate apex; in *P. maculata* the segment X arms are straighter and more slender. The female of *P. chipolensis* sp. nov. is similar to *P. maculata* and *P. lega* Ross in that all three species possess broad circular ventral plates on sternum VIII, but appears to be distinguishable by the shape and sclerotization of the internal structures of the vaginal apparatus. Additionally, *P. chipolensis* sp. nov. lacks a sclerotized dome-like band above the vaginal apparatus that is found in the other species [see Morse (1988: fig. 2A)].

###### Description.

Total length male 3.3–3.8 mm (mean = 3.6 mm, *n* = 10), female 3.2–4.0 mm (mean = 3.6 mm, *n* = 10). General structure typical of genus, as described by Robertson and Holzenthal (2013). In alcohol, head and thorax reddish brown; antennae, wings, and legs tawny; abdomen pale brown. Forewings with transverse translucent line at mid-length. Abdominal sterna clothed with long hairs; sternum VI with midventral keel-like projection.

**Figure 4.**
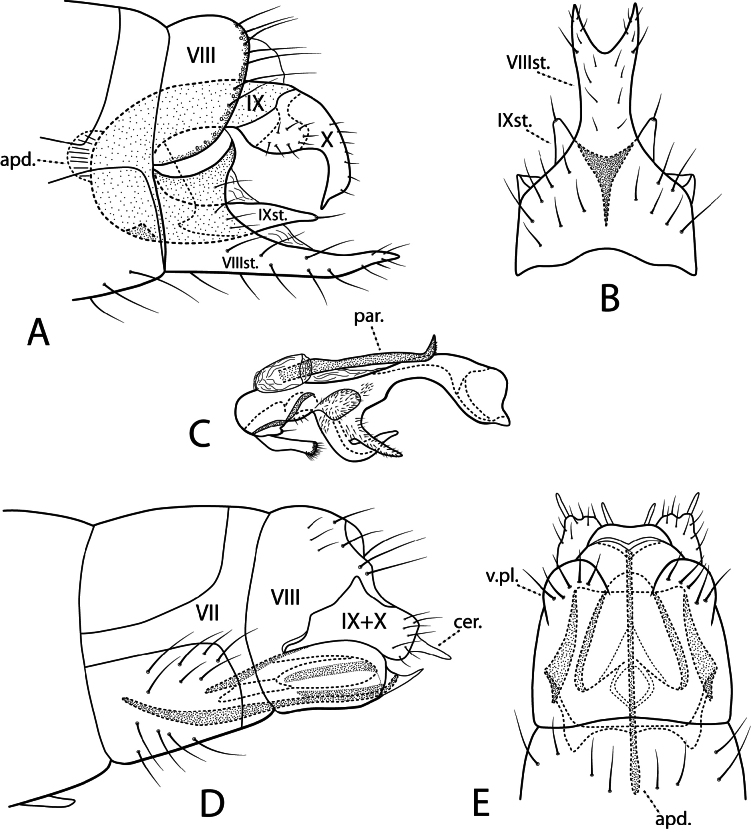
*Protoptila chipolensis* sp. nov. Male genitalia: **A** lateral **B** segment VIII ventral **C** phallic apparatus lateral. Female genitalia: **D** lateral **E** ventral. Abbreviations: apd. = apodeme; cer. = cercus; par. = paramere; st. = sternum; v.pl. = ventral plate.

***Male genitalia*.** Fig. 4A–C. Segment VIII tergum with darkened narrow band with long setae along posterior margin; in lateral view sternum dark brown basally, ventral margin straight, posterior projection elongate, slightly upturned apically; in ventral view sternum elongate, apex with rounded V-shaped mesal incision. Segment IX sclerotized, mostly enclosed within segments VII and VIII, sternum apically divided into pair of setal bearing processes. Segment X posteriorly divided into pair of curved arms; in lateral view bulbous basally with broad setal-bearing lobe, distally arm wide, decurved, cuspidate apically. Phallic apparatus nested within Segment IX attached to rounded apodeme; basoventrally with paired digitate rods, apically setose, appressible into pockets on underside of phallus; in lateral view pair of intertwined processes form ventral cup near midlength; pair of stout parameres attached to phallus dorsolaterally, in lateral view each paramere usually tucked underneath phallus, abruptly curved dorsomesally near apex, lightly spinous at tips; phallus in lateral view terminating in rounded head (phallicata) with posteroventral projection .

***Female genitalia*.** Fig. 4D, E. Segment VII unmodified, tergal and sternal plates rectangular. Segment VIII in lateral view dorsally rounded, constricted laterally, ventrally ovate with sharply pointed posteroventral projection; in ventral view ventral plates broadly rounded with erect black setae. Venter with vulvar lip slightly bilobed, posterior membranous area terminating in paired slender projections similar to cerci. Segment IX + X fused, posterolateral lobes broad; in lateral view lobes rectangular, slender cercus extending from apex; in ventral view lobes broadly conical with sinuous posterior margin. Internal apodeme “whip” attached ventrally near vulvar lip, extended cephlad far into Segment VII. Internal vaginal apparatus box-like in ventral view with circular spermathecal duct, paired elliptical structures, two pairs sclerotized lateral bands, in lateral view outermost band dorsal to inner band.

**Larva and pupa.** Undescribed. Larvae and a pupa of *P. chipolensis* sp. nov. have been collected and preserved for future study (see above material examined).

###### Distribution.

Known from only the Chipola River and tributaries in Jackson and Calhoun counties in the western panhandle of Florida. Collection records of the species from 14 sites were presented by Denson et al. (2016) as part of a faunal survey of the basin, which documented 143 species of Trichoptera.

###### Etymology.

This species is named after the Chipola River basin where the species was collected.

###### Remarks.

The Chipola River flows nearly 100 miles through northwest Florida emptying into the Apalachicola River. In many ways the Chipola River is atypical of most Florida rivers as it is fed by 63 freshwater springs which prevents water temperatures from becoming inhospitably warm in the summer. Portions of the river flow over limestone outcroppings, which is the primary larval habitat of the new species (Pescador et al. 2004) and *Setodes chipolanus* (Rasmussen et al. 2008b), which is also endemic to the Chipola River basin.

#### ﻿Integripalpia: Hydroptilidae

***Hydroptila* Dalman**

##### ﻿ Hydroptila aviforma

Taxon classificationAnimaliaTrichopteraHydroptilidae

Rasmussen & Harris sp. nov.

11D995E5-C36B-52F0-9DBE-AD4F5C0958F0

https://zoobank.org/5AA7F3A3-0496-4398-9163-12524BA936FC

Fig. 5

###### Type material.

***Holotype male*** (CMNH) • **Florida, Washington County**, Lucas Lake at boat landing, Lucas Lake Road, off County Road 279, 30°32'37"N, 85°41'26"W, 17 April 2013, A. Rasmussen and N. Miller, UV pan trap. ***Paratypes*** • Same data as holotype, 3 males (FAMU).

###### Diagnosis.

*Hydroptila aviforma* sp. nov. shares a number of characters with several southeastern species notably, *H. cretosa* Harris, *H. metteei*, and *H. wakulla*. All have an elongate tenth segment and inferior appendages which are equal in length in lateral view; a tenth tergite which is split distally; segment IX completely enclosed within segment VIII, which is tapered and rounded distally in lateral aspect; and a phallus which is narrow over nearly the entire length with the ejaculatory duct protruding apically. *Hydroptila aviforma* sp. nov. differs from these species in the tenth segment being wide and bulbous at midlength in lateral aspect, with a setal-bearing sclerite; the tenth tergite being deeply divided with each side thin and truncate distally; the inferior appendages having elongate, thick setae subapically; and segment VII lacking a ventromesal process.

###### Description.

**Figure 5.**
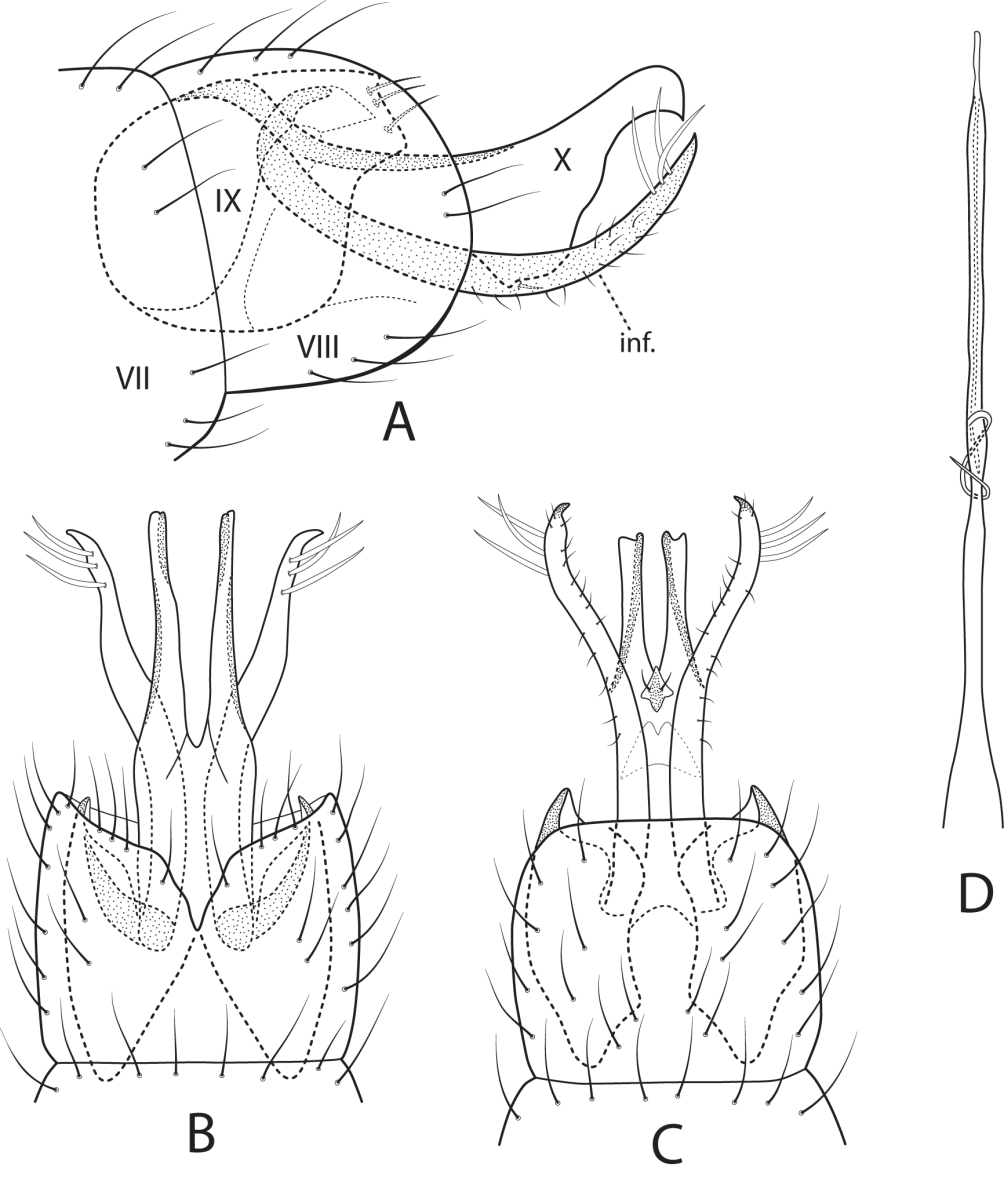
*Hydroptila aviforma* sp. nov. Male genitalia: **A** lateral **B** dorsal **C** ventral **D** phallus, ventral. Abbreviation: inf. = inferior appendage.

**Male.** Total length 2.8–3.0 mm (mean = 2.9 mm, *n* = 4). Antennae with 31 segments, brown in alcohol. Genitalia as in Fig. 5. Segment VII annular without a ventromesal process. Segment VIII wide anteriorly, rounded posteriorly in lateral view; dorsally incised on posteromesal margin; quadrate ventrally. Segment IX in lateral view short, incised posteroventrally, completely enclosed within segment VIII; dorsally divided mesally, posterolateral margins pointed distally; ventrally deeply incised on anterior margin. Segment X in lateral view widening at midlength, bulbous ventrally and bearing short, stout setae, tapering posteriorly to downturned pointed apex; dorsally deeply divided mesally, each lateral branch parallel-sided over length, truncate apically; ventrally with setal-bearing plate anterior of split. Inferior appendages thin and sickle-shaped in lateral aspect, bearing cluster of thickened, elongate setae posterodorsally, equal in length to segment X; in ventral view narrow and parallel-sided over much of length, sinuate posteriorly, pointed apically, with thickened setae subapically. Phallus elongate and thin over length, thin paramere encircling shaft below midlength, ejaculatory duct protruding apically.

**Adult female, larva, and pupa.** Unknown.

###### Distribution.

Known from only the type locality, a natural lake in the Florida panhandle.

###### Etymology.

Latin *avis* (bird) and *forma* (shape) referring to the bird-shape form of the apex of the tenth segment as seen in lateral view.

###### Remarks.

*Hydroptila quinola* (11 individuals) was the only other species of *Hydroptila* collected in the same sample as the new species. The addition of this new species brings the total number of Florida endemics in the microcaddisflies to 21, 14 of which are in the genus *Hydroptila*.

#### ﻿Integripalpia: Beraeidae

***Beraea* Stephens**

##### ﻿ Beraea jennyae

Taxon classificationAnimaliaTrichopteraBeraeidae

Rasmussen & Harris sp. nov.

E4F76AB9-0695-5F78-9E8C-10683883212D

https://zoobank.org/A19A020B-B2B6-4ACA-A924-343B75CEF9A6

Figs 6, 7

Beraea sp. nov.; original collection records, Rasmussen (2004).
Beraea sp. nov.; distributional record, Pescador et al. (2004).

###### Type material.

***Holotype male*** (CMNH) • **Florida, Okaloosa County**, Turkey Hen Creek, East branch steephead, Eglin Air Force Base, 0.3 km W of Okaloosa Lookout Tower, off SR-85, 30°38'48"N, 86°33'23"W, 8 April 1999, A. Rasmussen and M. Pescador, UV pan trap. ***Paratypes*** • Same data as holotype, 1 male (CMNH) • Same data as holotype except 9 April 1999, beating sheet, 1 female (CMNH) • Same data as holotype except 10 April 2001, 1 male (FAMU).

###### Diagnosis.

Of the North America species, male and female genitalia of *Beraea jennyae* sp. nov. are more similar to *B. fontana* Wiggins (Figs 8A–D, 9A–D) and *B. nigritta* Banks (Figs 8E–H, 9E–H) than to *B. gorteba* Ross (Figs 8I–L, 9I–L). Males of the new species are easily distinguished from the three other North American species by the lyre-shaped intermediate appendages, which are short, not reaching the apex of the X^th^ tergum; the intermediate appendages of *B. fontana* and *B. nigritta* in dorsal view are straighter and extend slightly beyond the X^th^ tergum, while in *B. gorteba* the intermediate appendages are long and sinuous in dorsal view extending well past the X^th^ tergum. Females of *Beraea jennyae* sp. nov. are differentiated from those of other North American species by the presence of a triangular, inward projecting process located along the midlength of each lateral lobe of the IX^th^ sternite.

###### Description.

**Figure 6.**
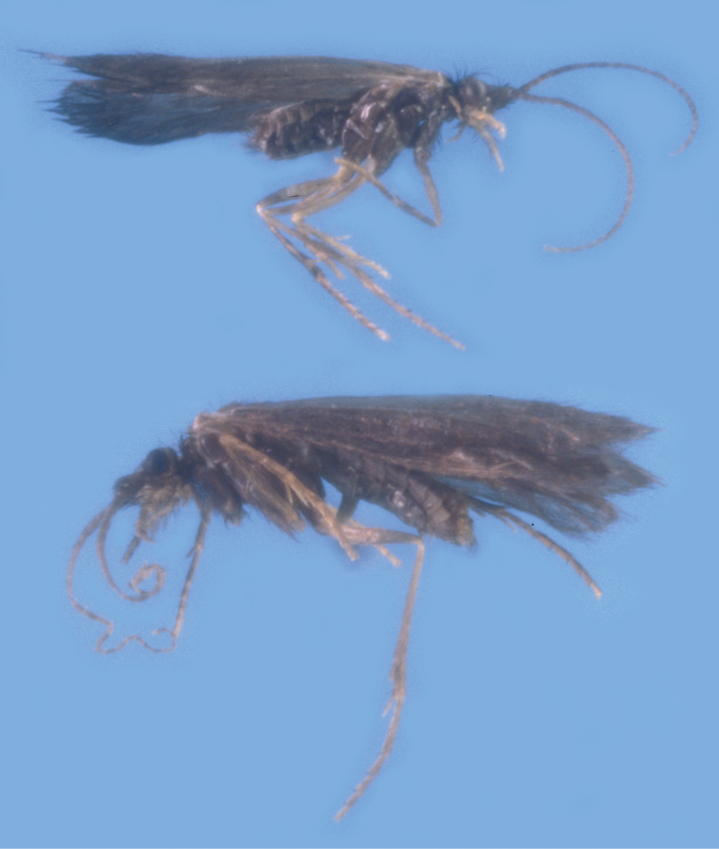
*Beraea jennyae* sp. nov. Photographs of adult female (top), male (bottom). Photographs by Peter Kovarik.

Fig. 6. Total length male 4.4–4.6 mm (mean = 4.5 mm, *n* = 3), female 4.0 mm (*n* = 1). In alcohol, body dark brown, legs somewhat lighter brown; wings light brown with dark brown hairs. General structure typical of the family and genus, as described by Ross (1944), Wiggins (1954), and Schmid (1998).

**Figure 7.**
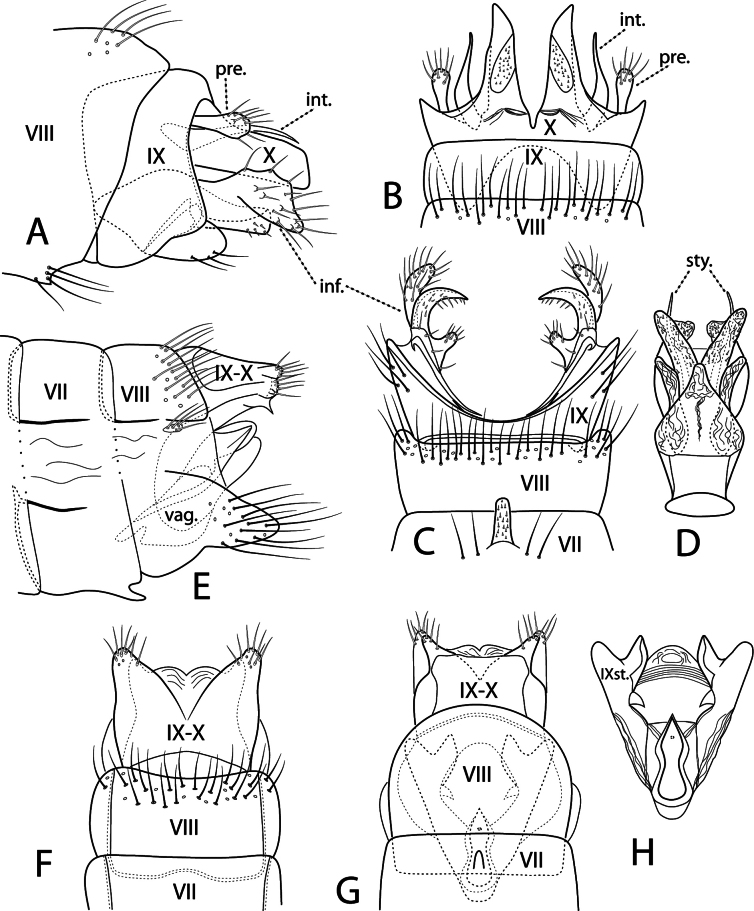
*Beraea jennyae* sp. nov. Male genitalia: **A** lateral **B** dorsal **C** ventral **D** phallus, ventral. Female genitalia: **E** lateral **F** dorsal **G** ventral **H** vaginal apparatus, ventral. Abbreviations: inf. = inferior appendage; int. = intermediate appendage; pre. = preanal appendage; sty. = style; vag. = vaginal apparatus; IXst. = segment IX sternum.

***Male genitalia*.** Fig. 7A–D. Segment VII annular, ventral mesal process elongate directed posteriorly. Segment VIII annular, posterior band of long setae dorsally and ventrally. Segment IX rectangular in lateral view; in ventral view posterior margin deeply concave. Preanal appendages clavate, about half length of intermediate appendages, stout setae posteriorly. Intermediate appendages in dorsal view lyre-shaped, curved outward basally, distally narrowed, terminating well short of Segment X apex. Segment X deeply incised dorsally forming two semi-membranous lobes, apex triangular with mesal margin angled outward; in lateral view rectangular, apex slightly rounded dorsally. Inferior appendages in lateral view base rectangular with membranous dorsal lobe directed posteroventrally, bearing many setae; in ventral view base narrow strap-like with curved mesal filament; stout circular hook posteriorly, with thumb-like basal process bearing setae. Phallus in ventral view thick, vasiform basally, single membranous lobe ventrally, paired lateral membranous lobes dorsally with pair of sclerotized styles running through middle terminating slightly beyond apex of lobes.

**Figure 8.**
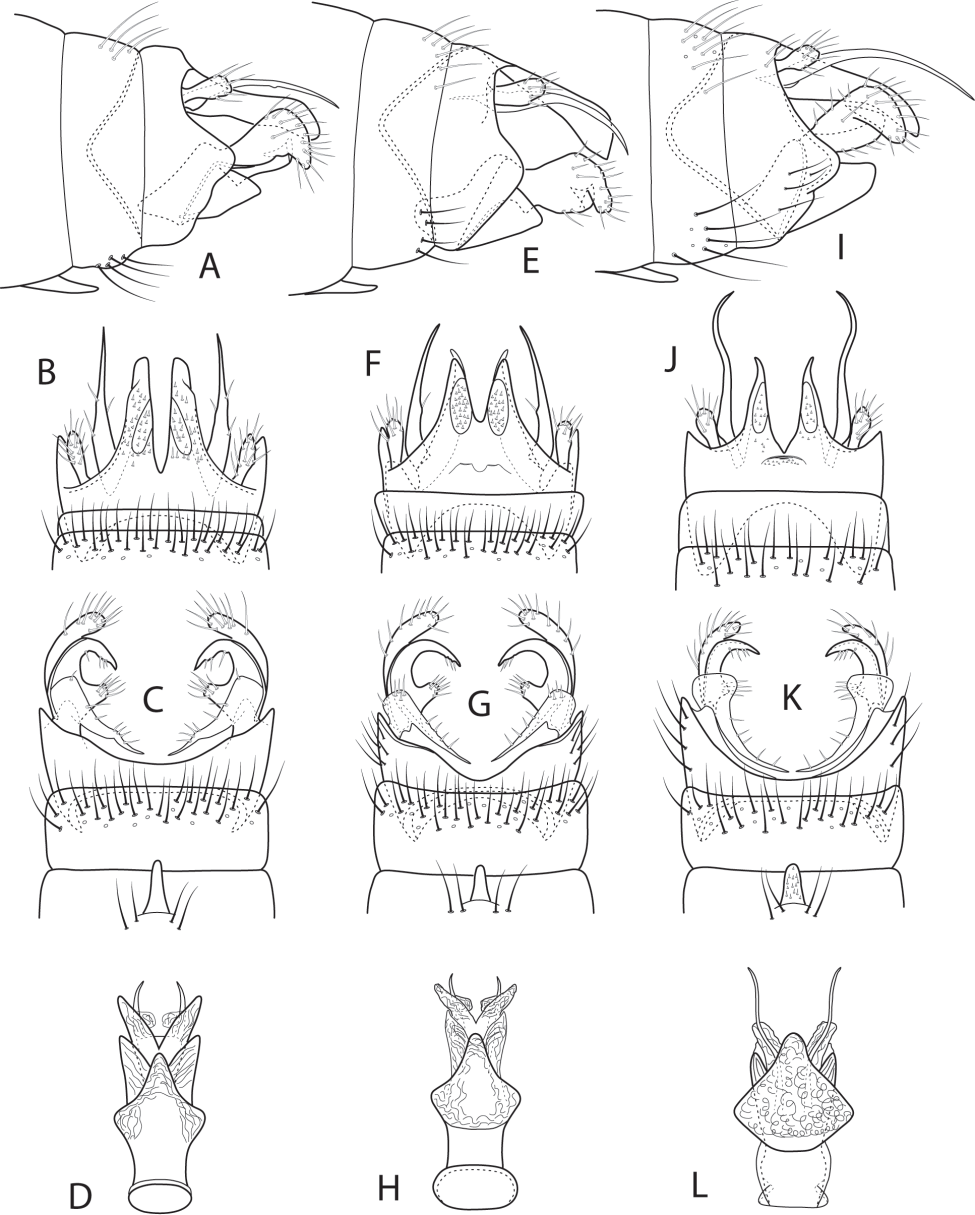
Male genitalia: *Beraea fontana***A** lateral **B** dorsal **C** ventral **D** phallus, ventral. *Beraea nigritta***E** lateral **F** dorsal **G** ventral **H** phallus, ventral. *Beraea gorteba***I** lateral **J** dorsal **K** ventral **L** phallus, ventral.

**Figure 9.**
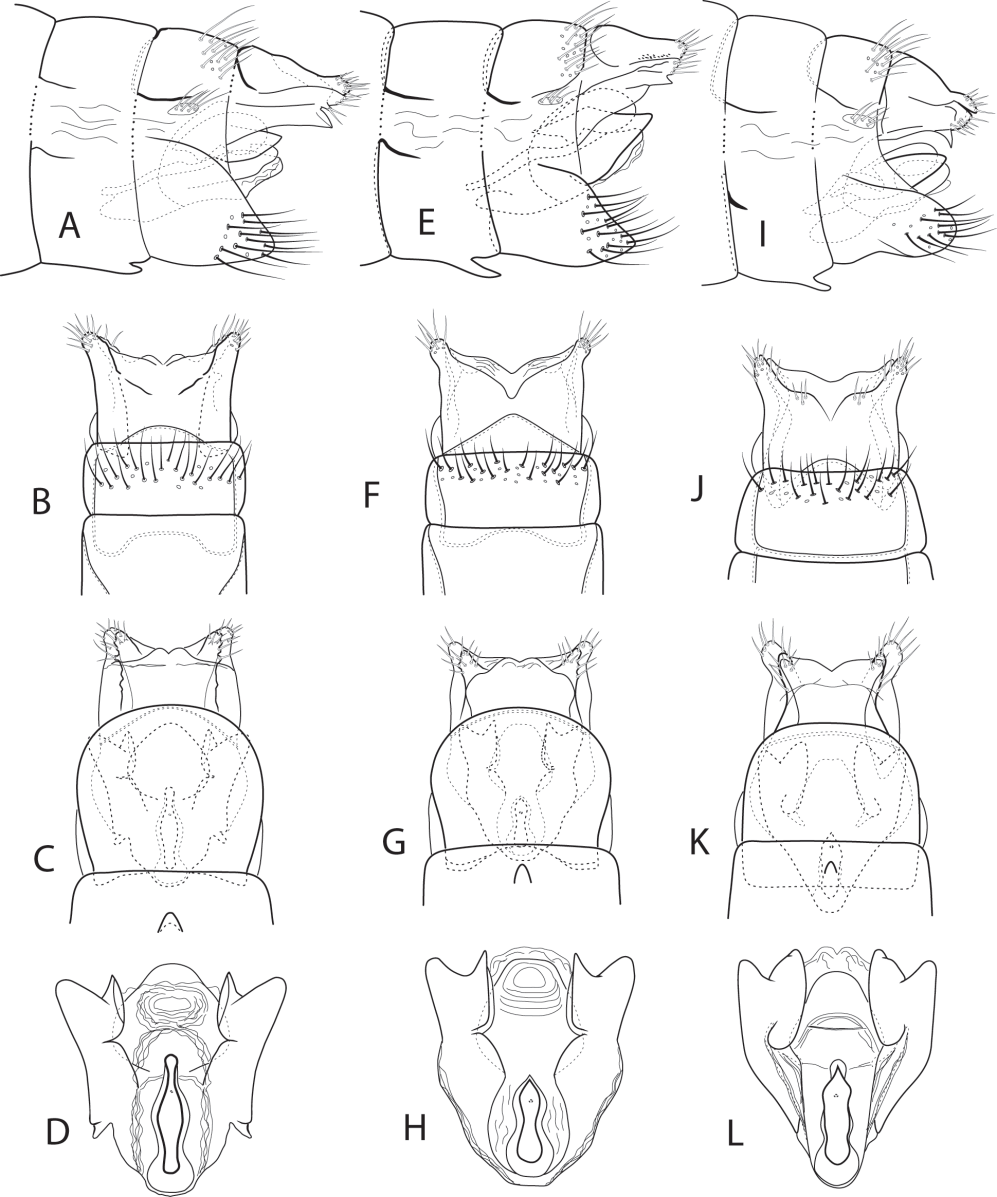
Female genitalia: *Beraea fontana***A** lateral **B** dorsal **C** ventral **D** vaginal apparatus, ventral. *Beraea nigritta***E** lateral **F** dorsal **G** ventral **H** vaginal apparatus, ventral. *Beraea gorteba***I** lateral **J** dorsal **K** ventral **L** vaginal apparatus, ventral.

***Female genitalia*.** Fig. 7E–H. Segment VII, VIII in lateral view with tergite and sternite separated by wide membranous pleuron, sternum VII with prominent mesal process directed posteriorly. Segment VIII tergum with posterior band of long setae; pleuron with small setal-bearing sclerite; sternum forming ventral flap covering vaginal apparatus in ventral view. Segments IX + X fused dorsally, apically with pair of posterolateral processes each side of anal opening, processes triangular in dorsal and ventral views, dorsal process broader than ventral process, ventral process in lateral view with posteroventral sharp point. Vaginal apparatus in ventral view with pair of sclerotized lobes (Segment IX sternites) lateral to genital chamber, apex of each lobe bifurcated with mesal process pointed, lateral process broadly rounded; triangular inward projecting process midlength along mesal margin of each sternal lobe; genital chamber with sclerotized ventral pouch 8-shaped, pointed posterior end.

**Larva and pupa.** Unknown.

###### Distribution.

The new species is known from only a single locality, a spring-fed ravine stream on Eglin Air Force Base in the western panhandle of Florida. Three other species are known in North America: *Beraea nigritta* and *B. fontana* each from a few localities, primarily in the northeast, and *B. gorteba* known from one stream in central Georgia.

###### Etymology.

This species is named for Xiaojing (Jenny) Wang, in appreciation of her love and support of the senior author throughout their marriage.

###### Remarks.

Specimens of adult males and females of the *Beraea fontana*, *B. gorteba*, and *B. nigritta* species were obtained for study from the U.S. National Museum, University of Minnesota Insect Collection, and the Clemson University Collection of Arthropods. Male and female genitalia for each species were illustrated (Figs 8, 9) and compared with *B. jennyae* sp. nov. These results may be used in a future review of the genus in North America.

#### ﻿Integripalpia: Leptoceridae

***Ceraclea* Stephens**

##### ﻿ Ceraclea pescadori

Taxon classificationAnimaliaTrichopteraLeptoceridae

Rasmussen & Harris sp. nov.

96E07090-1E8B-5FD2-85F7-58D6F19B6E36

https://zoobank.org/5E8A5E67-7EB7-4E4B-A213-824DDFEBF346

Figs 10, 11

Ceraclea sp. nov.; larval illustration, Pescador et al. 2004: fig. 168.
Ceraclea (Ceraclea) sp. nov. Glover, Carnagey, & Morse; description of female, Carnagey and Morse 2006: 29–30, fig. 28.
Ceraclea sp. nov. (nr. maculata); collection record, Denson et al. (2016).

###### Type material.

***Holotype male*** (CMNH) • **Florida, Washington County**: Lucas Lake at boat landing, Lucas Lake Road, off County Road 279, 30°32'37"N, 85°41'26"W, 17 April 2013, A. Rasmussen and N. Miller, UV pan trap. ***Paratypes*** • Same data as holotype, 10 males and 6 females (CMNH) • 10 males and 6 females (NMNH) • 10 males and 6 females (FAMU) • 10 males and 6 females (CUAC) • 5 males and 6 females (UMSP); **Calhoun County** • Page Pond at Page Pond Assembly of God, 30°32'21"N, 85°11'51"W, 7 May 2011, D . Denson, UV pan trap, 15 males (FAMU).

###### Diagnosis.

*Ceraclea pescadori* sp. nov. is placed in the Senilis group of Morse (1975) based on genitalic features of the male and female (Carnagey and Morse 2006). The male is most similar to *C. maculata*, but is easily distinguished by the more elongate tergum X and the presence of the much larger spinous, knob-like median lobe of the inferior appendages. Additionally, the basoventral lobe of the inferior appendages in ventral view is broadly triangular in *C. pescadori* sp. nov., versus thumb-like in *C. maculata*. The female is distinguished from other Senilis group members by the prominent and elongate sclerotic bulge and preanal appendages, and in the short wedge-shaped median plate (see also Carnagey and Morse 2006: fig. 28a, c).

###### Description.

Forewing length male 7.9–9.1 mm (mean = 8.3 mm, *n* = 10), female 7.7–8.1 mm (mean = 7.9 mm, *n* = 10). In alcohol, head and thorax reddish brown, abdomen light brown. Fore- and middle legs brown, hind legs light brown. Antennal scapes and pedicels brown, flagella light brown. Setal warts of head and thorax covered with primarily white hairs. Forewing R thickened near stigma, small translucent spots scattered anteriorly. Hind wings broad, unpatterned.

**Figure 10.**
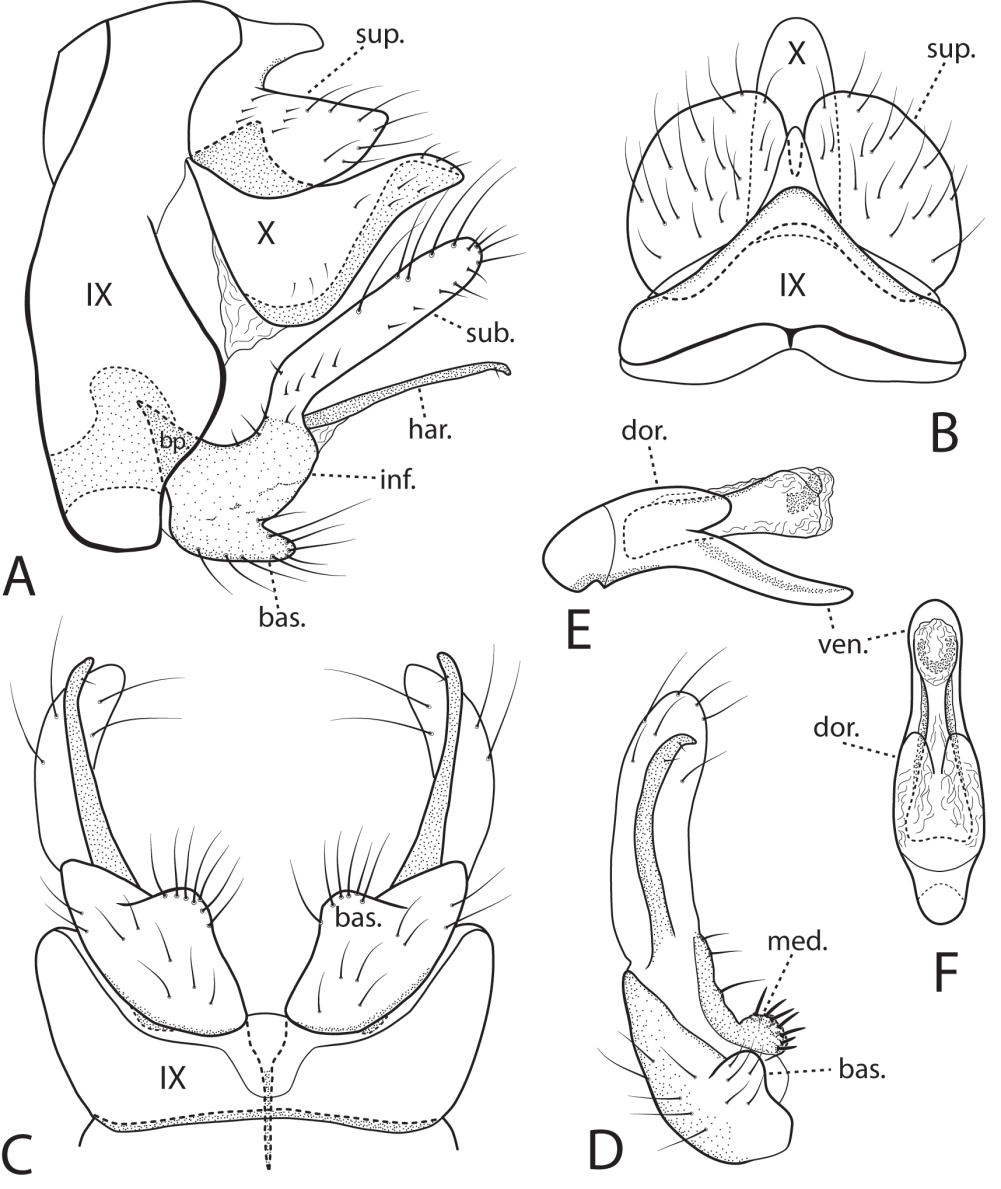
*Ceraclea pescadori* sp. nov. Male genitalia: **A** lateral **B** dorsal **C** segment IX and inferior appendages, ventral **D** inferior appendage, caudal **E** phallus, lateral **F** phallus, dorsal. Abbreviations: bas. = basoventral lobe; bp. = basal plate; dor. = dorsal lobe; har. = harpago; inf. = inferior appendage; sub. = subapico-dorsal lobe; sup. = superior appendage; ven. = ventral lip.

***Male genitalia*.** Fig. 10. Segment IX in lateral view narrow, rounded posteriorly; in ventral view excised mesally. Superior appendages in lateral view divided into dorsal beak-like process and larger ventral flap; in dorsal view dorsomesal process pointed, lateral lobes widely lobate. Segment X elongate dorsally, extended as undivided process well past superior appendages; in lateral view concave along dorsal margin, strongly convex along ventral margin. Inferior appendages in lateral view with acute triangular basal plate; basoventral lobes prominent, bearing long setae, in lateral view extending posteriorly, in ventral view broadly triangular, directed posteromesally; in caudal view with prominent, spinous median knob; subapical dorsal lobes elongate, straight in lateral view, curved slightly inward in ventral and caudal views, bearing short setae basally, mix of long and short setae distally; harpagos slender, nearly as long as subapical dorsal lobes, curved apically, bearing small setae subapically. Phallus phallobase with broad dorsal lobe extending to about mid-length of phallus, divided apically in dorsal view; in lateral view elongate ventral lip longer than phalicata; parameres absent.

**Figure 11.**
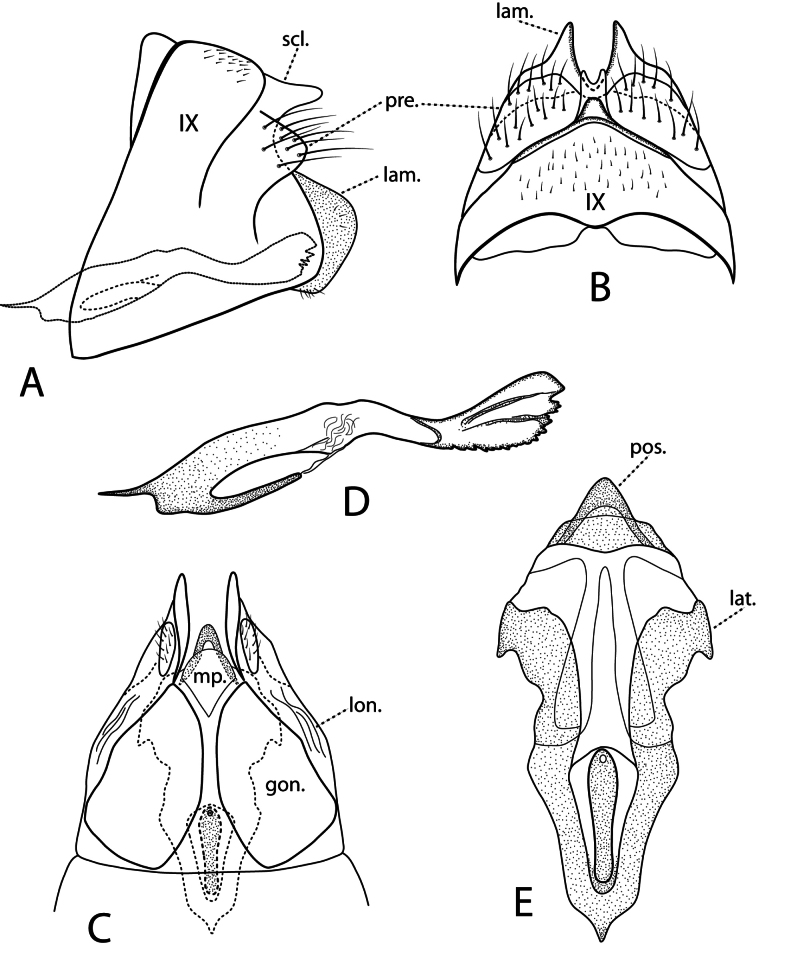
*Ceraclea pescadori* sp. nov. Female genitalia: **A** lateral **B** dorsal **C** ventral **D** spermathecal sclerite, lateral **E** spermathecal sclerite, ventral. Abbreviations: gon. = gonopod plate; lam. = lamella; lat. = lateral arm; lon. = longitudinal striations; mp. = median plate; pos. = posterior bridge; pre. = preanal appendage; scl = sclerotic bulge.

***Female genitalia*.** Fig. 11. Segment IX in lateral view rectangular, dorsal sclerotic bulge prominent; preanal appendages setaceous, posterior margin narrowly rounded. In dorsal view tergum IX arched, separated with creases from sclerotic bulge with median finger-like process usually bifid. Preanal appendages broad, fused mesally. Lamellae crescent shaped in lateral view; pointed in dorsal and ventral views. In ventral view, lateral gonopod plates isolated, anteriorly broad, tapered posteriorly; median plate with anterior half triangular, forming wedge between lateral plates, rounded posteriorly; longitudinal striations present posterolaterally. Spermathecal sclerite in ventral view generally vase-shaped, pointed anteriorly, deltoid sclerite absent, lateral arms somewhat sinuous, truncate posterolaterally, posterior bridge prominent, projecting past median plate; in lateral view acute anteriorly, clavate posteriorly with serrate posteroventral margin.

**Larva and pupa.** Illustrations of the head and pro- and mesonotum prepared by Dr James Glover were presented by Pescador et al. (2004: fig. 168). Pupa unknown.

###### Distribution.

This species is known from only two natural lakes located in the western Florida panhandle.

###### Etymology.

This species is named for Dr Manuel (Manny) Pescador in recognition of his contributions to trichopterology in Florida. His mentorship of the senior author and leadership of the aquatic entomology program at Florida A&M University are greatly appreciated.

###### Remarks.

This species was collected in the largest numbers from Lucas Lake, Washington County, Florida. Prior to this collection, Dr James Glover collected larvae and pupae of *Ceraclea* from Lucas Lake that he reared to adulthood in March/April of 2000. A preliminary description and figures of the male (Glover and Morse In Litt) confirm that the specimens collected by Glover represent the same species as we report here. Based on a single female pupa that was reared to adulthood by Glover, the female was described as *Ceraclea* sp. nov. (Carnagey and Morse 2006). Although we noted differences between their published description and the specimens we examined, especially in regard to the spermathecal sclerite, we contend that those differences are probably an artifact of lab rearing and specimen preparation or in interpretation, rather than the reared female specimen representing a different species. Also notable, single males of two other rare leptocerid caddisflies, *Ceraclea limnetes* and *Oecetis parva*, were collected in the same sample as the holotype.

#### ﻿Integripalpia: Leptoceridae

***Oecetis* McLachlan**

##### ﻿ Oecetis densoni

Taxon classificationAnimaliaTrichopteraLeptoceridae

Rasmussen & Harris sp. nov.

BA399CFD-7D61-5651-AFF1-771EF9897FAA

https://zoobank.org/3CC2461F-82B2-418D-B200-FF00169DC29D

Figs 12, 13

Oecetis sp. nov. (nr. cinerascens); collection records, Rasmussen et al. (2008a).
Oecetis sp. nov. (nr. cinerascens); collection records, Denson et al. (2016).

###### Type material.

***Holotype male*** (CMNH) • **Florida, Lake County**: Sellars Lake, Ocala National Forest, southeast lobe of lake, near canoe launch, 28 April 2007, D. Denson, A. Rasmussen, UV pan trap. ***Paratypes*** • Same data as holotype, 39 males, 1 female (CUAC) • unnamed lake, Ocala National Forest, jeep trail South off State Road 40, just East of Wildcat Lake, 17 May 2009, D. Denson, A. Rasmussen, UV pan trap, 84 males and 21 females (CMNH); **Marion County** • Lake Delancy, Ocala National Forest, Lake Delancy campground, 27 April 2007, D. Denson, A. Rasmussen, UV pan trap, 14 males and 1 female (USNM) • Fore Lake, Ocala National Forest, Fore Lake Recreation Area, 16 May 2008, D. Denson, A. Rasmussen, UV pan trap, 6 males (FAMU); **Calhoun County** • Wildcat Creek at State Road 20, 30°25'34"N, 85°08'34"W, 6 May 2011, D. Denson, 1 male (FAMU) • Page Pond at Page Pond Assembly of God, 30°32'21"N, 85°11'51"W, 7 May 2011, D. Denson, UV pan trap, 6 males (FAMU); **Jackson County** • Porter Pond at Pittman Hall Road, 30°35'17"N, 85°16'34"W, 20 May 2010, D. Denson, A. Rasmussen, UV pan trap, 1 male (FAMU); **Leon County** • Lofton Ponds, Apalachicola National Forest, at Sam Allen Road, 30°21'40"N, 84°23'25"W, 24 April 2007, M. Heyn, A. Wilson, UV-Light, 1 male (FAMU) • same as above except 25 June 2007, D. Denson, UV pan trap, 1 male (FAMU); **Liberty County** • Camel Pond, Apalachicola National Forest, Camel Pond campground, Forest Road 105, 30°16'37"N, 84°59'20"W, 16 May 2006, R. Flowers, A. Rasmussen, B. Richard, Mercury-vapor light, 3 males (UMSP); **Washington County** • Lucas Lake at boat landing, Lucas Lake Road, 30°32'37"N, 85°41'26"W, 17 April 2013, N. Miller, A. Rasmussen, UV pan trap, 4 males (UMSP).

###### Diagnosis.

*Oecetis densoni* sp. nov. belongs to the subgenus Pleurograpta, defined by Chen (1993) based on males having a very short IX^th^ tergum and long IX^th^ sternum. The new species is most similar to Oecetis (Pleurograpta) cinerascens in general appearance and shared characteristics of the male and female genitalia. The male of *O. densoni* sp. nov. and *O. cinerascens* differ from most species in the subgenus by the presence of one or no paramere spines in the phallus, versus many paramere spines typical of the subgenus. The new species differs from *O. cinerascens* in the absence of honeycomb sculpturing on the abdominal terga VI and VII and the dorsal margin of the inferior appendages having a less abrupt downturn midlength, as seen in lateral view. Females of both species have large circular gonopod plates with an anterior arcuate band of sclerotization and a posterior pair of concave sclerites. The new species female differs in having a more prominent mid-dorsal hump on segment IX and triangular preanal appendages versus in *O. cinerascens* a much less produced mid-dorsal hump on segment IX and less prominent, broadly rounded preanal appendages (in lateral view).

###### Description.

**Figure 12.**
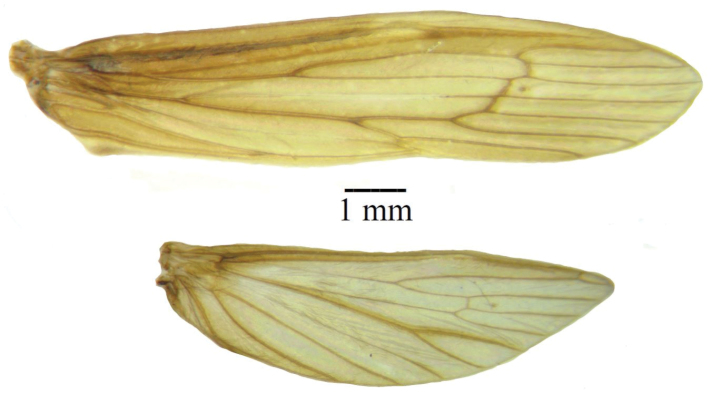
*Oecetis densoni* sp. nov. Wings, male: forewing, top; hind wing, bottom. Photographs by Alexander Orfinger.

Fig. 12. Forewing length male 8.4–10.2 mm (mean = 9.1 mm, *n* = 10), female 7.8–8.9 mm (mean = 8.1 mm, *n* = 10). Forewing with transverse base of MA and MP stem crossveins aligned diagonally at approximately 60^o^ angle, veins slightly darkened at forks. Nigma of fore and hind wings located near crossvein forks. In alcohol, head, wings, thorax, and legs reddish brown in males and grayish brown in females. Antennal scapes and pedicels reddish brown, flagella pale brown. Abdomen cream color, without honeycomb sculpturing on abdominal terga V–VIII.

**Figure 13.**
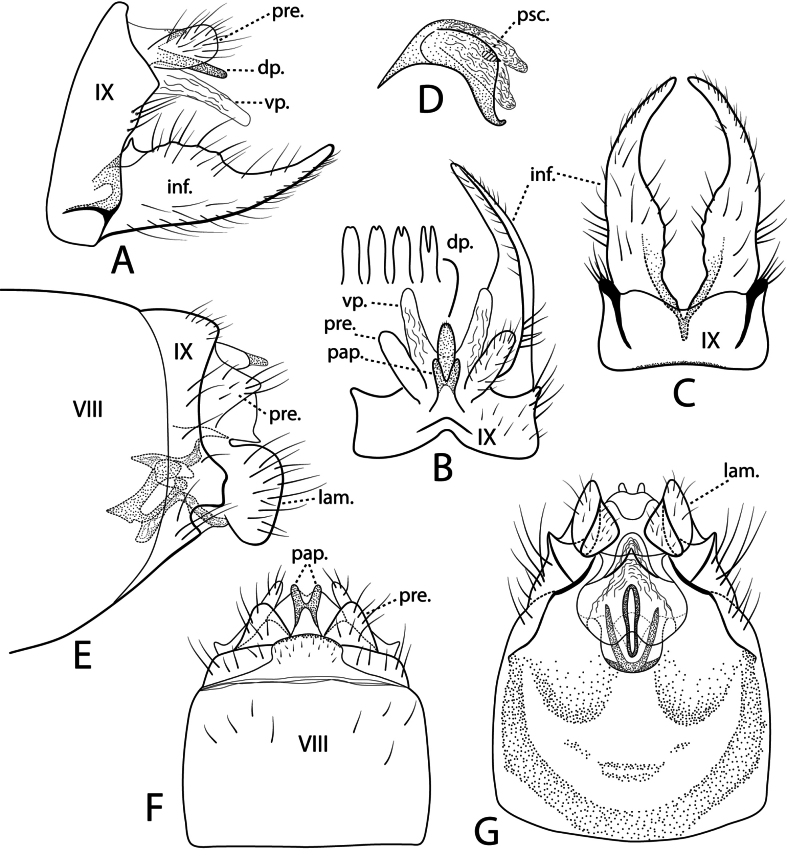
*Oecetis densoni* sp. nov. Male genitalia: **A** lateral **B** dorsal, showing variation in the dorsal process of segment X **C** segment IX and inferior appendages, ventral **D** phallus, lateral. Female genitalia: **E** lateral **F** dorsal **G** ventral, showing internal bursa copulatrix. Abbreviations: dp. = dorsal process of segment X; inf. = inferior appendage; lam. = lamella; pap. = papillae; pre. = preanal appendage; psc. = phallotremal sclerite; vp = ventral process of segment X.

***Male genitalia*.** Fig. 13A–D. Segment IX narrow dorsally, widened dorsolaterally, posterior margin somewhat angulate in lateral view, ventrally with thick line of sclerotization separating posterior sternal area; in dorsal view sclerotized papillae resembling rabbit ears posteriorly; in ventral view each posterolateral corner with line of heavy sclerotization diagonally directed anterad. Preanal appendages ovoid in lateral view, digitate in dorsal view, flared posterolaterally. Segment X consisting of sclerotized mid-dorsal process subequal in length to preanal appendages, subtended on each side by longer membranous ventral process; in lateral view dorsal process arising from under base of preanal appendage, ventral process curved posteroventally, far surpassing dorsal process; in dorsal view apical margin of dorsal process variable among populations, grading from entire to deeply incised. Inferior appendages symmetric; in lateral view base with dorsomesal point, dorsal margin irregularly scalloped anteriorly, posteriorly angled downward and smoothly tapered, apically upturned; in dorsal view ridge separating lateral area from excavated mesal area; in ventral view mesal margins scalloped, slightly concave, outer margins parallel basally, distally tapered, curved inward. Phallus without paramere spines; in lateral view phallobase sharply angled anteriorly, rectangular along mid-length, sharply downturned distally with lip upturned apically, tear drop-shaped phallotremal sclerite near ventral downturn of phallobase dorsal margin.

***Female genitalia*.** Fig. 13E–G. Segment IX in lateral view narrow, truncate posteroventral extension above concave emargination; in dorsal view broadly rounded median hump with rabbit ear-like papillae produced posteriorly; in ventral view posterolateral corners each with triangular projection with sclerotized line connecting apex to perpendicular sclerotized line at base. Preanal appendages prominent, subtriangular in both lateral and dorsal views. Lamellae ovate in lateral view; in ventral view each with ear-like projection. Gonopod plate (sternum IX) in ventral view large, subcircular with anterior arcuate band of sclerotization, posterior pair of concave sclerites. Bursa copulatrix [spermathecal sclerite of Chen (1993)] in ventral view with median elongate ring surrounded by deltoid sclerite anteriorly, posterior margin sclerotized forming inverted V.

**Larva and pupa.** Unknown. Based on the high similarity of adults of *Oecetis densoni* sp. nov. with *O. cinerascens* and the collection of the two species from similar lake habitats in Florida, the larva of the new species is likely to also be similar to *O. cinerascens*, as described and illustrated by Floyd (1995).

###### Distribution.

*Oecetis densoni* sp. nov. appears to be endemic to Florida’s clear lakes and ponds where its known geographic range extends from the Ocala National Forest northward into the Florida panhandle as far west as Washington County.

###### Etymology.

This species is named in honor of Dana R. Denson, who helped discover the species and has contributed greatly to the study and protection of Trichoptera biodiversity in Florida.

###### DNA Barcoding.

To visualize putative phylogenetic relationships, a Neighbor Joining (NJ) tree was constructed (Suppl. material 3) based on barcoding sequences of mitochondrial cytochrome oxidase I (COI) nucleotides using the built-in tree-builder of the Barcode of Life Database (BOLD) using Florida specimens examined in the course of this study. Based on this tree and morphological affinity, *O. cinerascens* (Hagen) is hypothesized to be the sister species of *Oecetis densoni* sp. nov. For *O. densoni* sp. nov. and *O. cinerascens*, pairwise divergence distances (p-distances) of COI sequences were calculated in MEGA11 (Tamura et al. 2021) using the Kimura 2-parameter evolution model (K2P) (Kimura 1980) and pairwise deletion of missing sites. Sequences were mined from BOLD and include both available *Oecetis densoni* sp. nov. sequences from specimens included in the present study (BOLDFLCAD120-09 and FLCAD140-09) as well as specimens of *O. cinerascens* from the Ocala National Forest (FLCAD130-09), northwest Florida (FLCAD148-09 and FLCAD056-08), and Virginia (PKCAD021-07), to account for population-level genetic variation. Nucleotide sequences were aligned using ClustalW using default settings (Larkin et al. 2007). All COI sequences used consist of 658 bp and were generated from vouchered specimens identified by taxonomic experts. Interspecific pairwise p-distance between *O. densoni* sp. nov. and *O. cinerascens* ranged from 6.4% to 6.9% (Suppl. material 2). These values are substantially larger than intraspecific p-distance ranges for *O. densoni* sp. nov. (0.08%) and *O. cinerascens* (0.2% to 0.5%), demonstrating a clear barcoding gap indicative of significant genetic differentiation between the congeners.

###### Remarks.

The presence of honeycomb sculpturing on abdominal terga V–VIII has been used to place Oecetis (Pleurograpta) species in the Testacea species group (Chen 1993). However, phylogenetic analysis by Quinteiro and Almeida (2021) of Neotropical *Oecetis* suggested that the Testacea species group is paraphyletic and that the honeycomb sculpturing of the abdominal terga probably originated more than once due to convergent evolution. In this study, the other Testacea species group members found in Florida, *Oecetis georgia* and *O. persimilis*, were shown to be closely related to each other (Suppl. material 3) but in a separate clade from *O. cinerascens* and *O. densoni* sp. nov. This is further evidence that honeycomb sculpturing is of limited use for placement of species into monophyletic groupings. Additionally, the presence of abdominal tergal sculpturing in *O. cinerascens* and the absence of it in *O. densoni* sp. nov, along with the relatively high degree of genetic and morphological similarities between the two species, further underscore the probable high evolutionary plasticity of abdominal tergal sculpturing in Trichoptera.

### ﻿Annotated checklist of Florida Trichoptera

#### ﻿Annulipalpians (fixed-retreat makers)

Table 1

**Table 1.** Checklist of Florida Trichoptera: Suborder Annulipalpia (fixed-retreat makers). Highlighted in bold font are species with the primary type specimen from Florida (see References for full citation of authorship). See Table 5 for definitions of conservation status rankings. † = status assessed by Rasmussen et al. (2008a). County records are also presented as a spreadsheet matrix in Suppl. material 1.

Taxon	Conservation status		County records
Dipseudopsidae			
*Phylocentropus carolinus* Carpenter, 1933	G5	–	1, 3–12
*Phylocentropus lucidus* (Hagen, 1861)	G5	–	8, 11, 12
*Phylocentropus placidus* (Banks, 1905)	G5	–	1–6, 8–16, 23–36
Hydropsychidae			
*Cheumatopsyche analis* (Banks, 1903)	G5	–	1–6, 8, 9, 11, 12, 14–19, 21–24, 27–30, 32–34, 36, 37, 40, 42, 43, 45, 47, 49, 51, 53, 54
*Cheumatopsyche burksi* Ross, 1941	G5	–	2, 3, 5, 10, 11, 14, 15, 22, 23, 28, 31–33, 35–37, 39–43, 45, 47–49, 51–53, 57–61, 63, 64
*Cheumatopsyche campyla* Ross, 1938	G5	–	8, 11, 12
*Cheumatopsyche edista* Gordon, 1974	G4	–	3–9, 11, 12, 25, 33
***Cheumatopsyche gordonae*** Lago & Harris, 1983	G1	S1^†^	2–4
*Cheumatopsyche minuscula* (Banks, 1907)	G5	–	8, 9
*Cheumatopsyche pasella* Ross, 1941	G5	–	1–9, 11–13, 17, 19, 24
***Cheumatopsyche petersi*** Ross et al., 1971	G3	S2^†^	1–4
*Cheumatopsyche pinaca* Ross, 1941	G5	–	1–3, 5–12, 14–19, 21–24, 28–37, 40, 42, 43
*Cheumatopsyche virginica* Denning, 1949	G5	–	1–4, 6, 8–14, 19, 23–25, 28, 32, 33, 36, 39, 40, 43, 48, 49, 53
*Diplectrona modesta* Banks, 1908	G5	–	1–4, 6–9, 11, 12, 14, 16, 24, 28
*Hydropsyche alabama* Lago & Harris, 1991	G1	S2	6, 8, 9
*Hydropsyche betteni* Ross, 1938	G5	–	9, 11, 12, 14, 16
*Hydropsyche decalda* Ross, 1947	G4	–	2–4, 6–9, 11, 12, 14, 16, 21, 24, 28, 32, 33, 43, 53
*Hydropsyche elissoma* Ross, 1947	G5	–	1–4, 6–12, 16, 24
*Hydropsyche incommoda* Hagen, 1861	G5	–	1–5, 7–16, 18, 20, 25, 32, 45, 57
*Hydropsyche mississippiensis* Flint, 1972	G5	–	1, 4–6, 8, 11, 13, 21
*Hydropsyche simulans* Ross, 1938	G5	–	1–5, 8–12, 14–23, 28, 30–33, 35, 36, 40, 43, 45, 47, 48, 51, 53, 57
*Hydropsyche* sp. in Lago & Harris, 2006	–	–	12
*Hydropsyche sparna* Ross, 1938	G5	–	1
*Macrostemum carolina* (Banks, 1909)	G5	–	1–15, 17, 19, 20, 23, 24, 32, 33, 35, 36
*Potamyia flava* (Hagen, 1861)	G5	–	8, 9, 11, 12
Philopotamidae			
*Chimarra aterrima* Hagen, 1861	G5	–	2–9, 11, 12, 14, 16, 18, 20, 21, 28, 32, 33, 36
*Chimarra falculata* Lago & Harris, 1987	G3	S1	1–4, 6–9, 11, 12
*Chimarra florida* Ross, 1944	G4	S3^†^	1–4, 6–13, 15, 17, 19, 21, 22, 24, 25, 27, 28, 30, 32–34, 37, 39, 40, 43, 45, 48, 49, 51, 53
*Chimarra moselyi* Denning, 1948	G5	–	1–5, 8–12, 14–23, 28, 30–33, 35, 36, 40, 43, 45, 47
*Chimarra obscura* (Walker, 1852)	G5	–	1, 4, 8–12, 23, 32
*Wormaldia moesta* (Banks, 1914)	G5	–	2–4, 8
Polycentropodidae			
*Cernotina calcea* Ross, 1938	G5	–	1–6, 8–25, 27, 28, 30–33, 35–38, 40, 41, 45, 47–49, 51, 58, 59, 61, 63, 64
*Cernotina spicata* Ross, 1938	G5	–	1–6, 8, 9, 11–17, 21, 24, 27, 28, 32, 33, 36, 37, 40–44, 47–49, 51, 52, 54, 57, 61, 64
***Cernotina truncona*** Ross, 1947	G4	S3^†^	2, 3, 6, 8, 9, 12, 14, 15, 24, 28, 32, 33, 36, 37, 40, 42, 43, 45, 48–50, 53, 59
*Cyrnellus fraternus* (Banks, 1905)	G5	–	1, 4, 5, 8–15, 17–25, 27–33, 35–38, 40–45, 47–53, 57, 58, 61, 63
*Neureclipsis crepuscularis* (Walker, 1852)	G5	–	1–12, 14–16, 18–24, 32, 33, 35, 36, 43, 45, 49, 53
*Neureclipsis melco* Ross, 1947	G4	–	1–4, 8–11
*Nyctiophylax affinis* (Banks, 1897)	G5	–	1–5, 7–9, 11, 12, 18, 24, 25, 28, 30–33
*Nyctiophylax celta* Denning, 1948	G5	–	1–4, 8, 9, 11, 32
***Nyctiophylax morsei*** Lago & Harris, 1983	G2	S2	2–4, 6, 7, 9, 12
*Nyctiophylax serratus* Lago & Harris, 1985	G4	–	1–12, 14–16, 18, 21–23, 25, 27, 28, 30, 32, 36, 37, 40, 45, 53
*Plectrocnemia cinerea* (Hagen, 1861)	G5	–	1–12, 14, 15, 20, 21, 24, 28, 31, 35, 36, 40, 49
*Plectrocnemia clinei* Milne, 1936	G5	–	3, 28
*Plectrocnemia crassicornis* (Walker, 1852)	G5	–	5, 8, 14, 23, 26
*Plectrocnemia nascotia* (Ross, 1941)	G5	–	3, 4, 6, 8, 9, 14, 36, 40, 42, 43, 49, 53
*Polycentropus blicklei* Ross & Yamamoto, 1965	G5	–	3, 4, 6, 9, 11, 12, 28, 33
*Polycentropus confusus* Hagen, 1861	G5	–	4
*Polycentropus elarus* Ross, 1944	G5	–	6
***Polycentropus floridensis*** Lago & Harris, 1983	G2	S2^†^	2–4
Psychomyiidae			
*Lype diversa* (Banks, 1914)	G5	–	1–12, 14, 20–24, 27, 28, 30–33, 35, 36, 40
*Psychomyia flavida* Hagen, 1861	G5	–	8, 9

The suborder Annulipalpia is represented in Florida by five families, 16 genera, and 50 nominal species. Although larvae of most species of fixed-retreat makers construct capture nets to filter food within lotic habitats, some warm-adapted members of the family Polycentropodidae due to their diverse adaptations are widespread throughout Florida in both lotic and lentic habitats.

##### Dipseudopsidae – pitot-tube caddisflies

The Florida dipseudopsid fauna consists of three of the five extant species within the genus *Phylocentropus* occuring in eastern North America. All members of the genus live in lotic habitats where the larvae reside in specialized “pitot” sand tube retreats buried in sandy depositional areas (Morse et al. 2019). *Phylocentropus placidus* occurs throughout North Florida, as far south as Marion County. The other two species, *P. carolinus* and *P. lucidus*, are restricted in Florida to the western part of the panhandle, with *P. lucidus* populations known in Florida from only small seepage streams within the Apalachicola/Chipola/Ochlockonee river basins.

**Hydropsychidae – net-spinning caddisflies**

Hydropsychid caddisflies are a major component of lotic macroinvertebrate communities of streams and rivers throughout Florida. Five genera and 21 species are reported in the state, with the most speciose genera, *Cheumatopsyche* (10 spp.) and *Hydropsyche* (8 spp.), represented across north and south Florida. The other three genera (*Diplectrona*, *Macrostemum*, and *Potamyia*) are represented in Florida by single species that are restricted to the northern half of the state. *Cheumatopsyche* (10 spp.) is one of the most commonly encountered genera of Trichoptera in Florida streams. The distributions and habitat associations of *Cheumatopsyche* species is diverse, with some species restricted to healthy streams and rivers of the western panhandle, and other species more widely distributed across the state and tolerant of a wide range of environmental conditions. *Cheumatopsyche petersi*, described from the Blackwater River in Florida, is endemic to the lower Gulf Coastal Plain (Rasmussen et al. 2008a). Other species restricted within Florida to the panhandle are *C. campyla*, *C. gordonae*, and *C. miniscula*. *Cheumatopsyche edista* and *C. pasella* are reported across the western panhandle and northern counties of the peninsula. More broadly distributed in the state, and recorded from diverse lotic habitats, are *C. analis*, *C. burksi*, *C. pinaca*, and *C. virginica*. *Diplectrona modesta* is reported from spring-fed headwater streams across the Florida panhandle eastward to Clay County. In the eastern United States, this species consists of several different haplotypes and associated larval morphotypes (Harvey et al. 2012). Rasmussen (2004) and Pescador et al. (2004) noted that the larval head coloration of specimens collected from Gold Head Branch (Clay Co.) was different from other *Diplectrona modesta* collected in Florida. DNA barcoding of Florida populations is needed, along with a broader revisionary study of the genus, to determine if *Diplectrona modesta* in Florida consists of a single variable species or cryptic species. *Hydropsyche*, like *Cheumatopsyche*, is represented by species inhabiting diverse lotic habitats across most of Florida. A species of special note is *Hydropsyche alabama*, described from Cowarts Creek in southeastern Alabama, is globally known from only the Chipola River basin and nearby Econfina Creek watershed. *Hydropsyche elissoma* is primarily restricted in Florida to healthy spring-fed sandhill streams. *Hydropsyche mississippiensis*, *H. incommoda*, and *H. simulans* (= *H. rossi* Flint, Voshell, & Parker) are more broadly associated with large streams and rivers, with the *H. incommoda* and *H. simulans* recorded from many panhandle and peninsular localities. *Hydropsyche sparna* is reported in Florida only from Brushy Creek, Escambia County (Rasmussen et al. 2008a). The species listed in Table 1 as *Hydropsyche* sp. was described by Lago and Harris (2006) based on two deformed male specimens within the *Hydropsyche scalaris* group that were collected from a spring-fed tributary of the Apalachicola River in Liberty County, Florida. The authors indicated that based on characteristics of the male genitalia, they believe the specimens probably represent a new species. However, due to its taxonomic uncertainty, *Hydropsyche* sp. was not included in the species totals presented here. *Macrostemum carolina* is known from most counties of northern Florida, as far south as Marion Co. The species occurs in a wide range of lotic habitats but is locally most abundant in large streams and rivers. *Potamyia flava* is a river species that in Florida is known from only the Apalachicola and Chipola river basins.

##### Philopotamidae – finger-net caddisflies

Philopotamids commonly occur in healthy streams and rivers throughout much of the state. The Florida fauna consists of two genera and six species: *Chimarra* (5 spp.) and *Wormaldia* (1 sp.). *Chimarra falculata*, endemic to the Southeastern Gulf Coastal Plain, is restricted in Florida to the western counties of the panhandle, while the other four *Chimarra* species (*C. aterrima*, *C. florida*, *C. moselyi*, *C. obscura*) are more widely distributed in eastern North American (Lago and Harris 1987) and more widespread in Florida, occurring in both the panhandle and peninsular Florida. *Wormaldia moesta*, distributed across much of eastern North America, reaches its southern-most limits in the Florida where it has been collected in healthy creeks within several counties of the western panhandle.

##### Polycentropodidae — trumpet-net caddisflies

Six genera and 18 species of polycentropodids are reported from Florida. Five of these genera include species that occur in diverse habitats throughout north and south Florida: *Cernotina* (3 spp.), *Cyrnellus* (1 sp.), *Neureclipsis* (2 spp.), *Nyctiophylax* (4 spp.), and *Plectrocnemia* (4 spp.); one genus, *Polycentropus* (4 spp.) is restricted to lotic habitats of north Florida. *Cernotina truncona* is endemic to the southeastern United States and occurs in north and south Florida, primarily in lakes and ponds (Rasmussen et al. 2008a, Orfinger and Bui 2024). *Cernotina calcea* and *C. spicata* are also widespread throughout the state, with *C. calcea* occurring primarily in lotic habitats and *C. spicata* associated with both lotic and lentic habitats. *Cyrnellus fraternus* is a common and widespread species in Florida, inhabiting a broad range lotic and lentic habitats. *Neureclipsis crepuscularis* and *N. melco* are lotic species, with *N. crepuscularis* reported across the panhandle and as far south as Highlands County on the peninsula. *Neureclipsis melco* is restricted in Florida to healthy creeks and rivers within the western panhandle counties. *Plectrocnemia* and *Polycentropus* are closely related genera, each represented in Florida by four species (Orfinger 2023). Of these, the most widespread and most often collected is *Plectrocnemia cinerea*, a common species in streams and rivers of the Florida panhandle and peninsula as far south as Osceola County (Rasmussen et al. 2008a). *Plectrocnemia crassicornis*, first reported in Florida (Jacksonville) by Banks (1907 as *P. australis* sp. nov.), has been collected from only a few other localities in north Florida with several collections coming from Black Creek in eastern Leon County. *Plectrocnemia clinei* is also rare in Florida and is known from only two localities in north Florida: Panther Creek in Okaloosa County, and Goldhead Branch in Clay County, reported by Rasmussen (2004). *Plectrocnemia nascotia*, first reported in Florida by Frost (1966) from Archbold Biological Station in Highlands County, has subsequently been collected from lakes and ponds of the panhandle and peninsula. The genus *Polycentropus* (*P. blicklei*, *P. confusus*, *P. elarus*, *P. floridensis*), is restricted in Florida to lotic habitats within the northern half of the state. *Polycentropus floridensis* is endemic to spring-fed streams of the lower Coastal Plain of Alabama and western part of the Florida panhandle (Rasmussen et al. 2008a). *Polycentropus blicklei* is recorded from a number of streams and creeks across north Florida, and *Polycentropus elarus* and *P. confusus* were recently recorded as new state records for Florida by Orfinger and Moulton (2021); each species being reported as a single collection from streams in the western panhandle.

##### Psychomyiidae – net-tube caddisflies

The psychomyiid fauna of Florida consists of the genera *Lype* and *Psychomyia*, each with only one species occurring in Florida. *Lype diversa*, the only North American species within the genus, has been collected from a wide range of stream types and sizes from counties across north Florida, as far south as Lake County. As is the case with *L. diversa*, *Psychomyia flavida* is widespread across eastern North America, but unlike *L. diversa*, *P. flavida* is known in Florida from only the Chipola River basin (Denson et al. 2016), occurring most abundantly in streams flowing over limestone outcroppings.

#### ﻿Integripalpians (basal lineages)

Table 2

**Table 2.** Checklist of Florida Trichoptera: Suborder Integripalpia (basal lineages). ⌂ = Florida endemic; * = new state record. Highlighted in bold font are species with the primary type specimen from Florida (see References for full citation of authorship). See Table 5 for definitions of conservation status rankings. † = status assessed by Rasmussen et al. (2008a); ‡ = status assessed by Rasmussen and Harris (2016). County records are also presented as a spreadsheet matrix in Suppl. material 1.

Taxon	Conservation status		County records
Glossosomatidae			
⌂ ***Protoptila chipolensis* sp. nov.**	–	–	8, 9
⌂ *Protoptila* sp. nov. (nr. palina)	–	–	6
* *Protoptila palina* Ross, 1941	G5	–	6
Hydroptilidae			
*Hydroptila acadia* Ross, 1941	GU	–	2, 4, 8, 9
*Hydroptila ajax* Ross, 1938	G5	–	1, 52, 57
*Hydroptila alabama* Harris & Kelley, 1984	G4	S2	1–3, 6, 8
⌂ ***Hydroptila apalachicola*** Harris et al., 1998	G1	S1	11, 12
*Hydroptila armata* Ross, 1938	G5	–	8, 9, 11, 12, 14, 15, 20, 23, 28, 32, 35, 36, 38, 41
⌂ ***Hydroptila auriscuspa*** Harris et al., 2012	GNR	–	3
⌂ ***Hydroptila aviforma* sp. nov.**	–	–	6
***Hydroptila berneri*** Ross, 1941	G4	S3	3, 4, 6, 8–10, 12, 14, 18–23, 28, 30–33, 35, 36, 40, 43, 45, 47, 49, 51, 52
⌂ ***Hydroptila bribriae*** Harris, 2002	G1	S1	2–4
*Hydroptila circangula* Harris, 1985	G2	S1	1–3, 7, 9
⌂ ***Hydroptila criokera*** Harris et al., 2012	GNR	–	12
*Hydroptila disgalera* Holzenthal & Kelley, 1983	G4	–	1–4, 6–9, 12, 15, 28
⌂ ***Hydroptila ebroensis*** Harris et al., 2012	GNR	–	2, 6, 7, 12
⌂ ***Hydroptila eglinensis*** Harris, 2002	G1	S1	2–4
*Hydroptila gunda* Milne, 1936	G5	–	8
*Hydroptila hamata* Morton, 1905	G5	–	3, 4, 8
⌂ ***Hydroptila hamiltoni*** Harris, 2002	G1	S1	3, 4
*Hydroptila icona* Mosely, 1937	G5	–	14
*Hydroptila latosa* Ross, 1947	G2	S2	2–4, 7–10, 16, 28
*Hydroptila lloganae* Blickle, 1961	G3	–	1–3, 11, 16, 19, 24, 28, 30, 32, 43, 45, 47–49, 52, 53
*Hydroptila maculata* Banks, 1904	G3	–	14, 15, 18, 23, 28, 31–33, 35, 36, 38, 40, 43, 47–50, 57, 59, 61, 67
*Hydroptila metteei* Harris, 1991	G1	–	8
***Hydroptila molsonae*** Blickle, 1961	G2	S2^†^	1–4, 6, 9, 12, 28, 43, 53
⌂ ***Hydroptila murtlei*** Harris et al., 2012	GNR	–	7, 9
*Hydroptila novicola* Blickle & Morse, 1954	G4	–	1–4, 9, 11, 12, 15, 24, 25, 27, 28, 43
⌂ ***Hydroptila okaloosa*** Harris, 2002	G1	S1	2, 3
*Hydroptila paralatosa* Harris, 1985	G2	S1	1, 8
*Hydroptila parastrepha* Kelley & Harris, 1983	G2	S1	1–3
*Hydroptila quinola* Ross, 1947	G5	–	1–9, 11, 12, 14–24, 27, 28, 30, 32, 33, 35, 36, 43, 49, 53
*Hydroptila remita* Blickle & Morse, 1954	G5	–	1–4, 6–9, 11, 12, 14, 28, 53
⌂ ***Hydroptila santarosa*** Harris et al., 2012	GNR	–	2
⌂ ***Hydroptila sarahae*** Harris, 2002	G1	S1	2–4
*Hydroptila scheiringi* Harris, 1986	G1	–	15
⌂ ***Hydroptila sykorai*** Harris, 2002	G1^‡^	S1	11
⌂ ***Hydroptila wakulla*** Denning, 1947	G2	S2^†^	14, 15, 32, 33, 36, 40, 47, 53
*Hydroptila waubesiana* Betten, 1934	G5	–	1–16, 28, 32, 48
*Hydroptila wetumpka* Harris, 1991	G1	S1	6
*Ithytrichia clavata* Morton, 1905	G5	–	8
*Ithytrichia* sp.	–	–	34
*Mayatrichia ayama* Mosely, 1937	G5		1–4, 8, 9, 11, 12, 15–17, 19, 20, 22, 23, 28, 31–33, 36, 40, 47, 57
*Neotrichia alabamensis* Kelley & Harris, 1983	G3	–	1, 3, 11, 12
***Neotrichia armitagei*** Harris, 1991	G2	S2	1–4, 6–12, 15, 24, 28, 32, 33, 49
*Neotrichia minutisimella* (Chambers, 1873)	G5	–	1–13, 18, 21, 22, 25, 28, 35, 53, 57
⌂ ***Neotrichia rasmusseni*** Harris & Keth, 2002	G1	S1	15, 20, 23, 28, 31, 32, 36, 40
*Neotrichia vibrans* Ross, 1938	G5	–	1–12, 14–17, 19–23, 27–33, 35–37, 39–43, 45, 47–49, 51, 52, 58, 61, 67
⌂ ***Ochrotrichia apalachicola*** Harris et al., 1998	GH	SX	3, 11, 12
*Ochrotrichia confusa* (Morton, 1905)	G5	–	12
⌂ ***Ochrotrichia okaloosa*** Harris, 1987 in Harris and Armitage (1987)	GH	SH^†^	3
⌂ ***Ochrotrichia provosti*** Blickle, 1961	GH	SH^†^	47
*Ochrotrichia tarsalis* (Hagen, 1861)	G5	–	1, 4, 15, 23, 32, 36, 40, 45, 47
*Orthotrichia aegerfasciella* (Chambers, 1873)	G5	–	1–9, 11, 12, 14–17, 19, 21–24, 27–30, 32, 33, 35–43, 45, 47–53, 55, 57, 59, 61, 63, 67
*Orthotrichia baldufi* Kingsolver & Ross, 1961	G5	–	11, 12, 30
*Orthotrichia cristata* Morton, 1905	G5	–	1–4, 6, 8, 9, 11, 12, 14, 15, 18, 21, 22, 24, 28, 29, 32, 36, 42, 50, 61, 66, 67
***Orthotrichia curta*** Kingsolver & Ross, 1961	G4	S2^†^	1, 3, 5, 6, 8, 9, 11–14, 16, 21, 23, 24, 28, 32, 36, 39–41, 43, 45, 47, 48, 53, 59
***Orthotrichia dentata*** Kingsolver & Ross, 1961	G2	S2^†^	1, 3, 4–6, 8, 9, 14, 15, 18, 20, 23, 28, 29, 31, 32, 36, 39, 42, 45, 47, 48
***Orthotrichia instabilis*** Denning, 1948a	G4	S4^†^	2, 3, 5, 6, 8, 9, 11–15, 24, 25, 32, 33, 36, 37, 39, 40, 42, 43, 53
*Oxyethira abacatia* Denning, 1947	G4	–	2–4, 6, 8, 9, 11, 12, 14, 15, 25, 27, 28, 36, 40, 43
*Oxyethira arizona* Ross, 1948	G3	–	47, 51, 52, 59
⌂ ***Oxyethira chrysocara*** Harris, 2002	G1	S1	6, 28
***Oxyethira elerobi*** (Blickle, 1961)	G3	S2^†^	1–4, 6, 7, 9, 12, 28
***Oxyethira florida*** Denning, 1947	G2	S2^†^	3, 28, 47, 67
*Oxyethira glasa* (Ross, 1941)	G5	–	1–4, 6–9, 11–16, 19–21, 23–25, 27–29, 32, 33, 36, 38–43, 45, 47–50, 53, 59, 61, 66, 67
*Oxyethira grisea* Betten, 1934	G5	–	12
***Oxyethira janella*** Denning, 1948a	G5	S4^†^	1–25, 27–33, 35–39, 41–43, 45, 47–49, 51–53, 57–59, 61
⌂ ***Oxyethira kelleyi*** Harris, 1987 in Harris and Armitage (1987)	G1	S1^†^	2–4
⌂ ***Oxyethira kingi*** Holzenthal & Kelley, 1983	GH	SH^†^	67
***Oxyethira lumosa*** Ross, 1948a	G4	–	2–9, 11–16, 18, 20–25, 27, 28, 30, 32, 33, 36–40, 43, 45, 47–49, 53
*Oxyethira maya* Denning, 1947	G5	–	2–4, 6, 8, 9, 11, 12, 14, 15, 17, 19–24, 27–33, 35–38, 40–43, 45, 47, 49–53, 57–59, 61, 67
*Oxyethira novasota* Ross, 1944	G4	S2^†^	1–5, 7–9, 11, 12, 14
*Oxyethira pallida* (Banks, 1904)	G5	–	1–9, 11, 12, 14–18, 21, 23, 24, 28, 32, 33, 36, 38–40, 42, 45, 47, 51, 52
*Oxyethira pescadori* Harris & Keth, 2002	G3	S3	1–4, 6–9, 11, 12, 14, 16, 18, 23, 24, 27, 28, 32, 33, 35, 36, 39, 40, 42, 43, 45, 49
*Oxyethira roberti* Roy & Harper, 1980	G3	–	1, 2, 6, 8, 9, 11, 14, 24, 41
*Oxyethira savanniensis* Kelley & Harris, 1983	G3	–	1–4, 6, 8, 9, 28
*Oxyethira setosa* Denning, 1947	G2^‡^	S2	2, 4, 6, 8, 9, 12
*Oxyethira simulatrix* Flint, 1968	–	–	5, 8, 20, 23, 29, 32, 35–38, 42, 43, 45, 47, 48, 51–53, 61, 63, 66
***Oxyethira sininsigne*** Kelley, 1981	G3	–	3, 6, 8, 12, 14, 15, 28, 36, 40, 53
*Oxyethira verna* Ross, 1938	G5	–	2, 4, 8, 11, 12, 15–19, 24, 28, 30, 32, 35–40, 42, 43, 45, 47–50, 53, 55, 59, 61, 67
*Oxyethira zeronia* Ross, 1941	G5	–	1–12, 14–16, 21–25, 28, 29, 32, 33, 35–37, 39–43, 45, 47–53, 59, 61, 66, 67
Rhyacophilidae			
*Rhyacophila carolina* Banks, 1911	G5	–	1–4, 7, 9, 11, 12
*Rhyacophila* sp. nov.	–	–	14

The basal lineages of the suborder Integripalpia are represented in Florida by three families, nine genera, and 82 species. Of the three families, the Hydroptilidae are by far the most diverse and widespread in Florida, represented by species distributed throughout the panhandle and peninsula in both lotic and lentic habitats.

##### Glossosomatidae – saddle-case caddisflies

Glossosomatids are known in Florida from only a three county area in the western panhandle, represented by a single genus *Protoptila* and three species. These diminutive caddisflies are especially common and abundant within the Chipola River basin where we report collecting *Protoptila chipolensis* sp. nov. from numerous sites in Jackson and Calhoun counties. Just west of the Chipola River basin in Washington Co., we record *P. palina* for the first time from Florida and report an undescribed *Protoptila* species from Econfina Creek, a calcareous, spring-fed system.

*Protoptila palina* Ross. **New State Record, Florida: Washington County** • Holmes Creek, Vernon at State Road 79, 30°37'38"N, 85°42'45"W, 21 July 1977, 1 male, D. Ray.

*Protoptila* sp. nov. (nr. palina). **Florida: Washington County** • Econfina Creek at Williford Spring, 50 m S of main spring, 30°26'20"N, 85°32'53"W, 12 May 2007, 2 males, UV-blacklight pan trap, A. Rasmussen • Econfina Creek at Walsingham Bridge Road, 30°28'57"N, 85°31'30"W, 5 June 2009, 60 males/females, UV-blacklight pan trap, A. Rasmussen, D. Ray, B. Albrecht, J. Bremer.

##### Hydroptilidae – microcaddisflies

The hydroptilids, often referred to as microcaddisflies, are the most taxa-rich family in Florida, with seven genera and 77 species reported in the state, including one species of *Hydroptila* new to science, *Hydroptila aviforma* sp. nov. Of the 77 Florida species, 21 species have been collected only in Florida, giving Hydroptilidae the highest percentage (27%) of Florida endemics among the four largest caddisfly families in the state. The two most speciose genera in Florida are *Hydroptila* (37 spp.) and *Oxyethira* (22 spp.), representing more than 75% of the microcaddisfly species in Florida, followed distantly by *Orthotrichia* (6 spp.), *Neotrichia* (5 spp.), *Ochrotrichia* (5 spp.), *Ithytrichia* (1 sp.), and *Mayatrichia* (1 sp.). Harris et al. (2012) presented an annotated checklist of the Hydroptilidae of Florida documenting the diversity and distribution of the 76 species known at the time. More recently, Harris and Rasmussen (2019) provided a review of the *Orthotrichia* species of Florida, with descriptions of previously unknown females for three species. County records presented in Table 2 include all the published county distributional data summarized by Harris et al. (2012), as well as additional county records from more recent collections. Notable recent unpublished collection records include *Hydroptila apalachicola* recovered from two new localities, namely Ocklawaha Creek (Gadsden Co.) and Bluff Creek (Liberty Co.), and larval collections of *Ithytrichia* sp. from Pringle Branch (Flagler Co.) in northern Florida near the east coast. The only previous report of *Ithytrichia* in Florida was *I. clavata* recorded from the Florida Caverns State Park (Jackson Co.) in the western panhandle by Moulton et al. (1999). *Oxyethira simulatrix* previously reported from numerous localities on the peninsula (Harris et al. 2012) has been recently collected from Holmes Creek (Holmes/Jackson county line) in the western panhandle.

##### Rhyacophilidae – free-living caddisflies

The Florida fauna of Rhyacophilidae contains the genus *Rhyacophila* represented by only one described species and one undescribed new species. This large genus, with 129 species reported from the United States and Canada (Rasmussen and Morse 2023), is most diverse in cool or cold streams and rivers, especially in mountainous areas. Not surprisingly, the two species known in Florida are restricted to the panhandle. *Rhyacophila carolina* has been recorded across the panhandle, primarily from small, spring-fed seepage streams, and *Rhyacophila* sp. nov. is recorded in Florida from two blackwater streams (Fisher Creek and Black Creek) in southern and eastern Leon County, respectively. The new species, closely related to *Rhyacophila dandaganu* Robinson & Parker and *Rhyacophila lobifera* Betten, also occurs in blackwater streams extending along the southeastern coastal plain to North Carolina. The new species will be described in a future publication in collaboration with Jason Robinson.

#### ﻿Integripalpians (portable tube-case builders)

Table 3

**Table 3.** Checklist of Florida Trichoptera: Suborder Integripalpia (portable tube-case makers). ⌂ = Florida endemic; * = new state record. Highlighted in bold font are species with the primary type specimen from Florida (see References for full citation of authorship). See Table 5 for definitions of conservation status rankings. † = status assessed by Rasmussen et al. (2008a); ‡ = status assessed by Rasmussen and Harris (2016). County records are also presented as a spreadsheet matrix in Suppl. material 1.

Taxon	Conservation status		County records
Beraeidae			
⌂ ***Beraea jennyae* sp. nov.**	–	–	3
Brachycentridae			
*Brachycentrus chelatus* Ross, 1947	G4	–	1–4, 8, 9, 11, 12
* *Brachycentrus lunatus* Harrington & Morse, 2004	G2	–	3
* *Brachycentrus nigrosoma* (Banks, 1905)	G4	–	11, 12
*Brachycentrus numerosus* (Say, 1823)	G4	–	1, 3, 4, 6, 7
***Micrasema florida*** Chapin & Morse, 2013	G2	S2	1–4
*Micrasema rusticum* (Hagen, 1868)	G5	–	1, 3, 7–9
*Micrasema wataga* Ross, 1938	G5	–	1–4, 7–9, 11, 12, 16, 20, 23, 28, 31–33, 35
Calamoceratidae			
*Anisocentropus pyraloides* (Walker, 1852)	G5	–	1–12, 14–16
*Heteroplectron americanum* (Walker, 1852)	G5	S2	2–4, 7, 8, 11, 12
Helicopsychidae			
*Helicopsyche borealis* (Hagen, 1861)	G5	–	4, 8, 9, 11, 14, 15, 19, 20, 23, 28, 31, 32, 36
Lepidostomatidae			
*Lepidostoma carrolli* Flint, 1958	G5	–	9
*Lepidostoma latipenne* (Banks, 1905)	G5	S1	11, 12
*Lepidostoma morsei* Weaver, 1988	G2^‡^	S1^†^	4, 12
*Lepidostoma wigginsi* Weaver, 2002	G3	S1	3, 11
Leptoceridae			
*Ceraclea cancellata* (Betten, 1934)	G5	–	1–15, 17–25, 28, 31–33, 35, 60
*Ceraclea diluta* (Hagen, 1861)	G4	–	1,3, 4, 7, 12
*Ceraclea flava* (Banks, 1904)	G5	–	3–5, 8–12, 17, 19–23, 31, 32, 35
⌂ ***Ceraclea limnetes*** Rasmussen & Harris, 2008 in Rasmussen et al. (2008b)	G2	S2	6, 8, 9, 14
*Ceraclea maculata* (Banks, 1899)	G5	–	1–19, 21–25, 27–30, 32, 33, 35, 36, 38, 40, 43, 45, 47–51, 53, 57, 61
⌂ ***Ceraclea pescadori* sp. nov.**	–	–	6, 9
*Ceraclea nepha* (Ross, 1944)	G5	–	1, 2, 4, 5, 8, 9, 11, 12, 15, 30, 32
*Ceraclea ophioderus* (Ross, 1938)	G5	–	1–6, 8–12, 14, 15, 21
*Ceraclea protonepha* Morse & Ross, 1975	G5	–	1–9, 11–15, 19, 30, 32
*Ceraclea resurgens* (Walker, 1852)	G5	–	2, 3, 8–10, 12, 13
***Ceraclea slossonae*** (Banks, 1938)	G4	–	46
*Ceraclea tarsipunctata* (Vorhies, 1909)	G5	–	1–4, 6–12, 14, 15, 19
*Ceraclea transversa* (Hagen, 1861)	G5	–	1–3, 5, 8–12, 14–16, 21, 23, 27, 28, 30, 32, 33, 36, 40, 43, 45, 47–49
*Leptocerus americanus* (Banks, 1899)	G5	–	5–12, 14–16, 20, 22–24, 27, 28, 30–33, 35, 36, 40–43, 45, 47, 51–53, 57, 58, 61, 64
***Nectopsyche candida*** (Hagen, 1861)	G5	–	1–5, 8–16, 19–23, 27, 28, 30–33, 35, 36, 40, 43, 47, 49, 54, 57
*Nectopsyche exquisita* (Walker, 1852)	G5	–	1–9, 11–15, 24, 28, 32, 33, 35, 36, 43, 45, 47–49, 51, 53
*Nectopsyche paludicola* Harris, 1986	G1	S1	1–4, 7
*Nectopsyche pavida* (Hagen, 1861)	G5	–	1–19, 21–25, 27–33, 35–38, 40–43, 45, 47–49, 51, 53, 54, 57, 61
*Nectopsyche spiloma* (Ross, 1944)	G5	–	1, 2, 9, 10, 12, 15, 35
⌂ ***Nectopsyche tavara*** (Ross, 1944)	G3	S3	25, 30, 32, 33, 35–38, 40–44, 48–51, 53, 58, 59, 63, 65, 67
*Oecetis avara* (Banks, 1895)	GNR	–	1, 8, 9, 15–21, 23, 28, 31, 32, 36
*Oecetis cinerascens* (Hagen, 1861)	G5	–	1–6, 8, 9, 11–19, 21–24, 26–30, 32, 33, 35–45, 47–54, 58, 59, 61, 63–65, 67
***Oecetis daytona*** Ross, 1947	G3	S2^†^	2–4, 10, 11, 21, 23, 24, 26, 28, 30, 32, 33, 37, 40, 42, 61
⌂ ***Oecetis densoni* sp. nov.**	–	–	6, 8, 9, 12, 14, 36, 40
*Oecetis ditissa* Ross, 1966	G5	–	1–4, 6–9, 11–16, 18, 22, 23, 25, 27, 28, 32, 39, 43, 45, 47, 52
*Oecetis georgia* Ross, 1941	G4	–	1–14, 16, 18, 19, 24–28, 32, 33, 36, 43, 53
*Oecetis inconspicua* (Walker, 1852)	G5	–	1–25, 27–33, 35–45, 47–54, 57–59, 61, 63–67
*Oecetis inconspicua* complex, sp. A in Floyd (1995)	–	–	1, 4, 45, 57, 58, 62
*Oecetis inconspicua* complex, sp. C in Floyd (1995)	–	–	14, 28, 32, 53
*Oecetis inconspicua* complex, sp. E in Floyd (1995)	–	–	14, 18
*Oecetis inconspicua* complex, sp. F in Floyd (1995)	–	–	3, 49, 53, 54, 60, 62
*Oecetis morsei* Bueno-Soria, 1981	G3	–	1, 3, 12, 33
*Oecetis nocturna* Ross, 1966	G5	–	1–5, 7–17, 19, 22, 24, 25, 27, 28, 33, 34, 42, 43, 49
*Oecetis osteni* Milne, 1934	G5	–	1–6, 8, 9, 11–19, 21, 23–25, 27, 28, 30–33, 35–37, 39–43, 45, 47–49, 51–53, 58
***Oecetis parva*** (Banks, 1907)	G2	S2^†^	6–9, 14, 15, 32, 33, 36, 39–41, 43, 49, 55
*Oecetis persimilis* (Banks, 1907)	G5	–	1–12, 14–24, 27, 28, 30–33, 35, 36, 40, 42, 43, 45, 47–49, 51, 53
***Oecetis porteri*** Ross, 1947	G3	S2^†^	6, 8, 9, 11, 12, 14, 23, 24, 27, 28, 32, 36, 37, 40, 42, 43, 48, 49, 53, 59, 67
⌂ ***Oecetis pratelia*** Denning, 1948b	GH	SH^†^	62
*Oecetis sphyra* Ross, 1941	G5	–	1–12, 14, 15, 19
⌂ ***Setodes chipolanus*** Rasmussen & Harris, 2008 in Rasmussen et al. (2008b)	G1	S1	8, 9
*Setodes guttatus* (Banks, 1900)	G4	S2	8, 9
*Triaenodes aba* Milne, 1935	G5	–	8, 12, 14, 16, 23, 32, 35, 36, 39, 41, 42, 47, 51, 53
*Triaenodes bicornus* Manuel, 2010	G2	S1	2–4, 11, 12, 28
***Triaenodes dendyi*** Manuel, 2010	G2	S1	3, 13, 14, 28, 33, 36, 40, 53
*Triaenodes flavescens* Banks, 1900	G5	–	15
***Triaenodes florida*** Ross, 1941	G3	S2^†^	5, 6, 8, 9, 12, 14, 15, 28, 32, 33, 35, 36, 40, 42, 53
⌂ ***Triaenodes furcella*** Ross, 1959	G3	S3^†^	6, 14–16, 20, 23, 28, 31–33, 35, 36, 40, 42, 45, 47, 49, 50, 53, 56, 58, 60, 62, 64, 66, 67
*Triaenodes ignitus* (Walker, 1852)	G5	–	1–12, 14–19, 21, 23, 26–28, 32–37, 40, 42, 43, 45, 47, 51, 53, 57
*Triaenodes injustus* (Hagen, 1861)	G5	–	8, 10, 42
⌂ ***Triaenodes lagarto*** Rasmussen & Harris, 2010 in Manuel (2010)	G1	S1	28
***Triaenodes milnei*** Manuel, 2010	GNR	–	1–6, 8–12, 14, 15, 19, 21–24, 26, 28–30, 32, 33, 36, 37, 39, 40, 42, 43, 45, 49, 51, 53
*Triaenodes nox* Ross, 1941	G5	–	14–16
*Triaenodes ochraceus* (Betten & Mosely, 1940)	G5	–	1, 3, 4, 8, 9, 11, 12, 14, 16, 26, 28, 32
*Triaenodes perna* Ross, 1938	G5	–	2–4, 6–12, 16, 24, 27, 28, 31, 33, 43
*Triaenodes smithi* Ross, 1959	G3	–	8, 12, 15
*Triaenodes taenia* Ross, 1938	G3	S1	11, 12
*Triaenodes tardus* Milne, 1934	G5	–	4, 7, 11, 12, 14, 15, 24, 33, 36, 41
Triaenodes cf. tardus in Manuel (2010)	–	–	12
*Triaenodes tridonta* Ross, 1938	G2^‡^	–	10
Limnephilidae			
*Ironoquia kaskaskia* (Ross, 1944)	G4	–	8
*Ironoquia punctatissima* (Walker, 1852)	G5	–	8, 14
*Pycnopsyche indiana* (Ross, 1938)	G5	–	3, 9, 14, 33, 43
*Pycnopsyche scabripennis* (Rambur, 1842)	G5	–	2–4, 8–12, 14, 16, 20, 24, 27, 28
Molannidae			
*Molanna blenda* Sibley, 1926	G5	–	3, 4, 6, 8, 9, 11, 12, 14, 16
*Molanna tryphena* Betten, 1934	G5	–	1–4, 8, 9, 11, 12, 14, 16, 18, 19, 24, 28, 30, 32, 34, 36, 40, 43
*Molanna ulmerina* Navás, 1934	G5	–	1–6, 8, 9, 12, 14, 15, 24, 28, 36
Odontoceridae			
*Psilotreta frontalis* Banks, 1899	G5	S1	3, 8, 11, 12, 14
* *Psilotreta labida* Ross, 1944	G5	–	8
Phryganeidae			
*Agrypnia vestita* (Walker, 1852)	G5	–	2, 3, 8, 12, 14, 19, 32
*Banksiola concatenata* (Walker, 1852)	G4	–	2–4, 11, 14, 16, 20, 24, 26, 32
*Ptilostomis ocellifera* (Walker, 1852)	G5	–	2–4, 8–10, 12, 14, 16, 28
*Ptilostomis postica* (Walker, 1852)	G5	–	2–6, 8–12, 14, 16, 20, 24, 32
Sericostomatidae			
*Agarodes crassicornis* (Walker, 1852)	G5	–	2–4, 6, 7, 9, 11, 12, 28
*Agarodes griseus* Banks, 1899	G4	–	11
*Agarodes libalis* Ross & Scott, 1974	G3	S3^†^	1, 3, 4, 6–9, 11, 12, 14–16, 24, 28, 32, 36
⌂ ***Agarodes logani*** Keth & Harris, 1999	G1^‡^	S1	11
*Agarodes wallacei* Ross & Scott, 1974	G1	–	3
⌂ ***Agarodes ziczac*** Ross & Scott, 1974	G2	S2^†^	2–4

The portable tube-case builders of the suborder Integripalpia are represented in Florida by 11 families, 21 genera, and 88 nominal species and four larval morphotypes within the *Oecetis inconspicua* complex. Ten of the 11 families are restricted to lotic habitats in the northern half of the state, with only the warm-adapted family Leptoceridae having species that occur in both lentic and lotic habitats across the panhandle and peninsula.

##### Beraeidae – spring-loving caddisflies

The beraeids are a small family represented in North America by four species within the genus *Beraea*; one species, *Beraea jennyae* sp. nov., is reported from Florida. This Florida endemic is known only from a few adult specimens collected from one steephead ravine stream in Okaloosa County (Rasmussen 2004). In North America, *Beraea* species occur only in eastern North America, with populations that are extremely localized and restricted to small spring fed streams in which larvae inhabit wet organic muck of seepage areas (Hamilton 1985; Wiggins 1996).

##### Brachycentridae – humpless caddisflies

The brachycentrid fauna of Florida comprises seven species placed within the genera *Brachycentrus* (4 spp.) and *Micrasema* (3 spp.). All of these species are restricted in Florida to lotic habitats (creeks and rivers) in the western panhandle, with the exception of *Micrasema wataga* which also occurs in the northern portion of the peninsula where it has been collected in abundance in rivers such as the Santa Fe. Of the *Brachycentrus*, *B. chelatus* is the most widespread and commonly collected species, while the other two species (*B. lunatus* and *B. nigrosoma*) we report for the first time in Florida from the sites recorded below. Of special note: *Micrasema florida*, is endemic to western parts of the Florida panhandle and coastal Alabama; the holotype specimen was collected from the Blackwater River in Okaloosa Co., Florida (Chapin and Morse 2013).

*Brachycentrus lunatus* Harrington & Morse. **New State Record, Florida: Okaloosa County** • Honey Creek, Eglin Air Force Base at Range Road 211, 30°41'38"N, 86°29'45"W, 13 February 2013, 1 male, D. Ray.

*Brachycentrus nigrosoma* (Banks). **New State Record, Florida: Gadsden County** • Crooked Creek at County Road 270, 30°34'58"N, 84°53'02"W, 6 May 2004, dipnet, 1 larva, M. Pescador, A. Rasmussen, B. Richard • same data, except, 22 July 2005, R. Flowers, M. Pescador, A. Rasmussen, B. Richard; **Liberty County** • Sweetwater Creek at County Road 270, 30°31'58"N, 84°58'03"W, 13 January 2012, beating sheet, 3 males, A. Rasmussen, A. Heupel.

##### Calamoceratidae – comb-lipped caddisflies

Despite Calamoceratidae having the highest diversity in tropical and subtropical regions of the world, the Florida fauna due to its geographic isolation from the Neotropics contains only the Nearctic genus *Heteroplectron*, represented in eastern North America by *H. americanum*, and the primarily southeastern species *Anisocentropus pyraloides*. Both species are restricted in Florida to small spring-fed streams of the panhandle, but *A. pyraloides* is fairly common as compared to *H. americanum*, which is restricted to cool-water refugia such as steephead ravine streams.

##### Helicopsychidae – snail-case caddisflies

One helicopsychid species is reported in Florida, *Helicopsyche borealis*, a widespread and common species across much of North America. Within Florida, *H. borealis* has been collected primarily, and most abundantly, from calcareous creeks, rivers and large spring runs across the panhandle and on the peninsula as far south as the Rainbow River in southwestern Marion County. In a revision of the *Helicopsyche* of the Americas, Johanson (2002) noted that Florida populations of *H. borealis* exhibit significant morphological differences in the male genitalia that merit further study and analysis.

##### Lepidostomatidae – bizarre caddisflies

More than any other family in Florida, the lepidostomatids exemplify a cool-adapted fauna with disjunct southern populations occurring in cool-water refugia of the western panhandle. The Florida fauna consists of the genus *Lepidostoma* represented by four species. Among the species are *L. morsei*, a lower Gulf Coastal Plain endemic (Weaver 1988; Rasmussen and Harris 2016), and three species (*L. carrolli*, *L. latipenne*, *L. wigginsi*) that are widespread in the eastern United States. *Lepidostoma carrolli* is known in Florida from a single collection made in Calhoun County (Denson et al. 2016), while *L. latipenne* is reported in Florida from only spring-fed ravine streams within the Apalachicola River basin (Rasmussen 2004), and *L. morsei* and *L. wigginsi* are known from spring-fed ravine streams in western and eastern counties of the panhandle.

##### Leptoceridae – long-horned caddisflies

Generally speaking, leptocerids are the most common, abundant, and diverse portable tube-case builders inhabiting lotic and lentic habitats throughout the state. More than 40% of the leptocerid species recorded in the United States occur in Florida where we report 54 nominal species placed within 6 genera. Species richness of the various genera ranged as follows: *Leptocerus* (1 sp.), *Setodes* (2 spp.), *Nectopsyche* (6 spp.), *Ceraclea* (13 spp.), *Oecetis* (15 spp.), and *Triaenodes* (17 spp.). *Leptocerus americanus*, the sole North American species within the genus, is common throughout much of the state in association with waters having submerged aquatic plants. The genus *Setodes*, unlike the other genera which are widespread in Florida, is known only from the Chipola River basin where the narrow-range endemic *Setodes chipolanus* and the eastern species *S. guttatus* are both reported (Rasmussen et al. 2008b; Denson et al. 2016). *Nectopsyche* is a diverse genus in Florida in terms of species geographic distributions and habitat types. *Nectopsyche pavida*, the smallest and most common *Nectopsyche* in Florida, occurs throughout the state in wide range of lotic and lentic habitats. *Nectopsyche tavara*, endemic to the Florida peninsula, occurs primarily in lakes of the central Florida highlands (Daigle and Haddock 1981), and *N. paludicola* is endemic to streams of the lower Gulf Coastal Plain of Alabama and western counties of the Florida panhandle. *Nectopsyche candida* and *N. exquisita* are similar species (Ross 1944; Glover and Floyd 2004) reported from streams and rivers in both the panhandle and peninsula, with *N. candida* being the most common. Records of *N. exquisita*, particularly from the peninsula, are questionable and likely apply to *N. candida*. *Nectopsyche spiloma* has been infrequently collected, most often from panhandle rivers. The genus *Ceraclea* is represented in Florida by six species groups as defined by Morse (1975) comprising widespread Nearctic species, along with two species endemic to lakes of the Florida panhandle, namely, *C. limnetes* (Fulva group) and *C. pescadori* sp. nov. (Senilis group). *Ceraclea maculata* and *C. cancellata*, both members of the Senilis group, are widespread throughout Florida with *C. maculata* being the most often collected *Ceraclea* in the state. Other Fulva group members occurring in Florida are *C. resurgens* from the western panhandle counties and *C. transversa*, which occurs in a variety of lotic and lentic habitats across the panhandle and the peninsula as far south as Polk County in central Florida. *Ceraclea tarsipunctata*, *C. nepha*, and *C. protonepha*, all members of the Tarsipunctata group, occur in creeks and rivers of the panhandle and northern part of the peninsula. *Ceraclea flava* (Riparia group) and *C. ophioderus* (Nigronervosa group) are recorded mainly from large creeks and rivers of the panhandle and northern part of the peninsula. *Ceraclea slossonae* (Nigronervosa group), described in 1938, is known in Florida only from the type material collected from Belleair near the Gulf coast in Pinellas County. *Ceraclea diluta* (Diluta group), a diminutive species, is restricted in Florida to streams and rivers of the western panhandle. The genus *Oecetis* is more than any other genus of portable tube-case builder, the most ubiquitous and prevalent genus recorded from lotic and lentic habitats statewide. Within Florida we report 15 nominal *Oecetis* species and four larval morphotypes (not included in the species total) described by Floyd (1995) that represent potential cryptic species within the *Oecetis inconspicua* complex. The following summary of Oecetis species utilizes the subgenus taxonomic classification provided by Chen (1993). The subgenus Pleurograpta is represented in Florida by four species (*O. cinerascens*, *O. densoni* sp. nov., *O. georgia*, *O. persimilis*). *Oecetis cinerascens*, widespread across much of North America, is also widespread throughout Florida with records from a variety of lentic and lotic habitats within 54 counties. In contrast, the closely related *O. densoni* sp. nov. is endemic to Florida and restricted to natural lakes of the panhandle and peninsula. *Oecetis georgia* and *O. persimilis* are closely related lotic species recorded from numerous streams and rivers throughout the state as far south as Highlands County. The subgenus Quaria is represented in Florida by two species (*O. morsei*, *O. sphyra*). Both lotic species, adults of *O. sphyra* have been light-trapped in abundance from many large streams and rivers throughout the panhandle. Interestingly, records of *O. morsei* we have from the panhandle were single specimens identified within samples containing large numbers *O. sphyra*. The close morphological similarity of the two species and co-occurrence suggests to us that these Florida specimens may represent a morphological variant of *O. sphyra* and may not be a distinct species. Of note, Floyd (1995) described both species as larvae and found that they are indistinguishable. The subgenus Oecetis is represented in Florida by five nominal species (*O. ditissa*, *O. inconspicua*, *O. nocturna*, *O. porteri*, *O. pratelia*) and four larval morphotypes described by Floyd (1995) within the *O. inconspicua* complex (sp. A, sp. C, sp. E, sp. F). *Oecetis ditissa* and *O. nocturna* are reported from many streams and rivers of the panhandle and the northern half of the peninsula. *Oecetis inconspicua* is the most common and widespread caddisfly species within Florida with collection records from diverse lentic and lotic habitats within 60 counties. Morphological variation within *O. inconspicua* observed by researchers suggests that *O. inconspicua* comprises a complex of closely related species, yet to be resolved. Additionally, the species complex hypothesis is supported by research conducted by Floyd (1995) involving rearing of *Oecetis* specimens to adulthood that resulted in the descriptions of distinct larval morphotypes with adults identified to be *O. inconspicua*. While Floyd’s larval morphotypes are not definitive evidence of cryptic species, we believe that it is important to recognize this “hidden” biodiversity within the genus and have therefore included the larval morphotypes in the checklist. Larvae of *Oecetis inconspicua* complex sp. A were recorded from several lotic habitats in south Florida and western panhandle, and *Oecetis inconspicua* complex sp. C, sp. E, and sp. F were recorded primarily from lentic habitats. *Oecetis porteri* is a lentic species with scattered collection records from lakes throughout the state. *Oecetis pratelia*, known based only on the type specimen collected in 1939 from Hendry Co., may be extinct (Floyd 1995; Rasmussen et al. 2008a). The Oecetis subgenus Pseudosetodes is represented in Florida by four species (*O. avara*, *O. daytona*, *O. osteni*, *O. parva*). *Oecetis avara*, widespread across parts of North America, is recorded across the northern half of the state, primarily from calcareous rivers (Blahnik and Holzenthal 2014). *Oecetis osteni* is recorded from lentic and lotic habitats statewide as far south as Glades County, with highest recorded abundances coming from natural lakes. *Oecetis daytona* is a rare coastal plain endemic that in Florida has been infrequently recorded from lotic habitats primarily in the northern half of the state (Rasmussen et al. 2008a). *Oecetis parva*, also a coastal plain endemic, is a minute species reported from ponds and lakes across northern and central Florida (Burington et al. 2011). The *Triaenodes* fauna of Florida comprises a diverse mix of coastal plain endemics and more widespread Nearctic species. The following summary of the *Triaenodes* fauna of Florida utilizes the taxonomic classification provided by Manuel (2010). *Triaenodes aba*, *T. dendyi*, and *T. nox*, all members of the subgenus Triaenodes, are recorded in Florida from lentic and lotic habitats, with *T. aba* and *T. dendyi* reported from localities scattered over much of the state and *T. nox* recorded from only few locations grouped in a three county area of the panhandle. Larvae of all three species are known to live on aquatic plants (Glover 1996). *Triaenodes milnei* and *T. perna* (Perna group) are relatively common species throughout much of Florida with larvae found in association with stream-side underwater roots of woody vegetation (Glover 1996); a few records of *T. milnei* are also from tannic lakes on the peninsula. *Triaenodes ochraceus* (Dipsius group) occurs primarily in blackwater streams of the panhandle and northern peninsula, with larvae likely inhabiting cypress roots. *Triaenodes ignitus* (Ignitus group) is by far the most widespread and common species of *Triaenodes* in Florida, occurring in a wide range of lotic habitats throughout the state. Other Ignitus group members in Florida are *T. bicornis*, *T. florida*, *T. taenia*, and *T. lagarto*. *Triaenodes bicornis* is reported from streams and creeks in a few scattered locations across northern Florida. Its sister species, *T. florida*, is a strictly lentic species associated with aquatic macrophytes in ponds and lakes of the panhandle and peninsula (Glover 1996). *Triaenodes taenia*, a species of the southern Appalachians, has disjunct populations occurring in spring-fed ravine streams within Gadsden and Liberty counties (Rasmussen 2004); its close relative, *T. lagarto*, is known from only the type specimens collected from Alligator Creek, a lake run stream within Clay County. Three other extremely rare species in Florida are *T. flavescens* and *T. smithi*, both members of the Flavescens group, and *T. tridonata* (Tridonta group). *Triaenodes flavescens* is known in Florida based only on larval collections from the St. Marks River, Wakulla County, and *T. smithi* is recorded from single creeks in Jackson and Wakulla counties and reported from Liberty County by Manuel (2010). *Triaenodes tridonta* is known in Florida from only a single adult male collected in 1927 from Dead Lakes, Gulf County (Rasmussen and Harris 2016). *Triaenodes furcella* (Injustus group) is a Florida endemic that has been recorded from lentic and lotic habitats across the peninsula and into panhandle Florida as far west as Washington County. *Triaenodes injustus*, a common and widespread species across much of North America, is rare in Florida and has been recorded from only a few streams in the Chipola River basin (Denson et al. 2016) and from Gulf and Orange counties by Manuel (2010). *Triaenodes tardus* (Marginatus group), also widespread and common in North America, is reported in Florida from a variety of lentic and lotic habitats in the panhandle and as far south as Hernando County on the peninsula. Manuel (2010) reported that this species is quite variable and included illustrations and description provided by Rasmussen and Harris of a male Triaenodes cf. tardus that was collected in Florida from Kennedy Creek in western Liberty County. Manuel (2010) indicated that this specimen may or may not represent a distinct species and that additional material (males and females) is needed to make this determination.

##### Limnephilidae — northern caddisflies

The limnephilid fauna of Florida comprising *Ironoquia* (2 spp.) and *Pycnopsyche* (2 spp.) represents a very small subset of the taxa-rich limnephilds found in eastern North America. Florida limnephilids emerge as adults primarily in the fall, unlike the springtime emergence of most other caddisfly species in Florida. *Ironoquia* has been infrequently collected in Florida and appears to be restricted to the panhandle. Denson et al. (2016) reported the only known occurrence of *I. kaskaskia* in Florida from the Chipola River, Jackson County. *Ironoquia punctatissima* is known also in Jackson County from the Chipola River and several of its tributaries, as well as from the Ochlockonee and St. Marks river basins in Leon County. *Pycnopsyche scabripennis* [= *P. antica* (Walker)] and *P. indiana* are fairly common in healthy streams and creeks across northern Florida and have been reported as far south on the peninsula as Seminole County where *P. indiana* was recorded by Rasmussen and Denson (2000).

##### Molannidae – hood caddisflies

This small and distinctive family, represented in Florida by three species within the genus *Molanna*, is an important component of macroinvertebrate communities of healthy streams and rivers of northern Florida. *Molanna blenda* is restricted to small streams of the panhandle, in particular seepage-fed ravine streams (Rasmussen 2004). In contrast, *Molanna tryphena* and *M. ulmerina* are more widespread and common, with occurrences reported from a wide variety of streams and rivers across northern Florida as far south as Seminole and Marion counties, respectively.

##### Odontoceratidae – mortarjoint caddisflies

Odontocerids are uncommon in Florida and represented by two species within the genus *Psilotreta*, including *P. labida* which is recorded here (see below) for the first time from the state. The other species, *Psilotreta frontalis*, occurs in small spring-fed seepage streams of the panhandle, with most collection records coming from Gadsden and Liberty counties (Parker and Wiggins 1987; Rasmussen 2004).

*Psilotreta labida* Ross. **New State Record, Florida: Jackson County** • Mill Creek, West of Maddox Road, 30°36'52"N, 85°14'00"W, 24 August 2020, D-frame dipnet, 1 larva/case, R. Frydenborg.

##### Phryganeidae – giant caddisflies

Phryganeids are much more common and diverse in northern latitudes than in Florida where we report only four species placed in three genera: *Agrypnia* (*A. vestita*), *Banksiola* (*B. concatenata*), and *Ptilostomis* (*P. ocellifera*, *P. postica*). All four of these species are uncommon in collections and have been reported from scattered localities across the panhandle and northern part of the peninsula (Wiggins 1998; Denson et al. 2016). Specimen collection localities indicate a range of habitat types from wet prairies and ponds to streams and rivers.

##### Sericostomatidae – bush-tailed caddisflies

The sericostomatid fauna of Florida comprises six of the 12 species recognized within the eastern North American genus *Agarodes* (Keth and Harris 2008). The genus is most diverse in the southeast and the Florida fauna contains two species endemic to the state: *Agarodes ziczac* endemic to streams of the western part of the Florida panhandle (Rasmussen et al. 2008a) and *Agarodes logani* known from only a single spring-fed ravine stream in Gadsden County (Rasmussen and Harris 2016). *Agarodes crassicornis* and *A. libalis* are relatively widespread and common species that occur in clean streams and creeks of the panhandle and northern part of the peninsula. Far less common are *A. wallacei*, which we report based on specimens collected on several occasions (1979–1981) from the Blackwater River in Okaloosa County, and *Agarodes griseus*, which we report in Florida from females collected at the type locality of *A. logani*.

### ﻿Erroneous or dubious records from Florida

Table 4

**Table 4.** Selected species with literature records from Florida that are erroneous or dubious. The references listed are the primary literature sources that recorded the species in Florida.

Taxon	Reference	Remarks
*Ceraclea floridana* (Banks, 1903)	Banks (1903); Morse (1975); Carnagey and Morse (2006)	*Nomen dubium*. Known from only the female holotype collected in south Florida along Biscayne Bay. Examined by Morse (1975), he noted that the abdomen was missing.
*Ceraclea spongillovorax* (Resh, 1974)	Gordon (1984); Carnagey and Morse (2006)	The only reported occurrence was Leon Co. Florida by Gordon (1984); record erroneous according to Pescador et al. (2004).
*Cheumatopsyche sordida* (Hagen, 1861)	Frost (1966); Burington (2011)	Frost reported this species from Archbold Biological Station, Highlands Co., based on 3 females. We believe this record is doubtful and that the specimens are probably *C. virginica* Denning.
*Chimarra parasocia* Lago & Harris, 1987	Pescador et al. (1995)	Reported erroneously; however, the species possibly occurs in Florida based on occurrences in Gulf Coastal eastern Louisiana and Alabama.
*Chimarra socia* Hagen, 1861	Betten (1934); Ross (1944)	Ross (1948b) stated that records of *C. socia* for Florida, Georgia, and Illinois in Ross (1944) should be deleted because they apply to *Chimarra moselyi* (= *C. perigua* Ross).
*Hydatophylax argus* (Harris, 1869)	Mattson et al. (1995)	The Mattson et al. (1995) record is questionable and is likely based on misidentified larval *Pycnopsyche*.
*Hydropsyche phalerata* Hagen, 1861	Flint et al. (1979)	Flint et al. (1979) reported *H. phalerata* from Florida. Flint re-examined these specimens and determined them to be *H. alabama* (O.S. Flint, pers. comm, 2010). The specimens were collected 4 May 1970 from the Florida Caverns State Park (Jackson Co.).
*Hydropsyche scalaris* Hagen, 1861	Betten (1934)	Record is dubious; Betten (1934) reported the species from “Bainbridge, Fla., July 16”. Bainbridge is a city in southwest Georgia.
*Hydroptila strepha* Ross, 1941	Frost (1966)	Probable misidentifications. *Hydroptila* specimens Frost collected from Archbold Biological Station, Highlands Co. were examined and listed as paratypes in the description of *Hydroptila morsei* by Sykora and Harris (1994), a junior synonym of *H. lloganae* Blickle (Etnier and Baxter 1999).
*Holocentropus interruptus* Banks, 1914	Gordon (1984)	Doubtful or erroneous state (Walton Co.) record according to Pescador et al. (2004).
*Lepidostoma griseum* (Banks, 1911)	Pescador et al. (1995, 2004); Rasmussen (2004); Rasmussen et al. (2008a)	Misidentifications. Specimens were reexamined and determined to be *L. morsei* Weaver by Rasmussen and Harris (2016).
*Nectopsyche albida* (Walker, 1852)	Banks (1892); Gordon (1984)	Doubtful or erroneous state record (Levy Co.) according to Pescador et al. (2004).
*Neophylax concinnus* McLachlan, 1871	Pescador et al. (1995)	Doubtful or erroneous state record according to Pescador et al. (2004).
*Neotrichia okopa* Ross, 1939	Hamilton and Schuster (1978); Blickle (1979); Keth et al. (2015)	Dubious state record according to Harris et al. (2012).
*Nyctiophylax moestus* Banks, 1911	Armitage and Hamilton (1990); Pescador et al. (1995)	Doubtful or erroneous state record according to Pescador et al. (2004).
*Pycnopsyche guttifera* (Walker, 1852)	Gordon (1984)	Erroneous report of misidentified *Pycnopsyche scabripennis* [= *P. antica* (Walker)] according to Pescador et al. (2004).
*Triaenodes helo* Milne, 1934	Manuel (2010)	According to Manuel (2010)*T. helo* is a hybrid form of *T. perna* Ross x *T. milnei* Manuel. He did not report examination of any specimens of *T. helo* from Florida (nearest report from Mobile Co., Alabama). The specimens previously reported as *T. helo* from Florida are correctly applied to *T. milnei*, although *T. helo* is possible in Florida where *T. perna* and *T. milnei* co-occur.

### ﻿Conservation status of Florida Trichoptera

Table 5

**Table 5.** Conservation status rankings of the caddisfly species listed in Tables 1–3 as assigned by NatureServe (2024).

Global status	No. species	Florida status	No. species
G1 (critically imperiled)	20	S1 (critically imperiled)	27
G2 (imperiled)	21	S2 (imperiled)	24
G3 (vulnerable)	21	S3 (vulnerable)	7
G4 (apparently secure)	25	S4 (apparently secure)	2
G5 (secure)	112	SH (possibly extirpated)	4
GH (possibly extinct)	5	SX (presumed extirpated)	1
GNR or GU (unranked)	8		

The global conservation status rankings by NatureServe (2024) of the caddisfly species listed in Tables 1–3 indicate that 112 of 220 species are considered secure (G5) and 25 species are apparently secure (G4). There were roughly equal numbers of species ranked as critically imperiled G1 (20 spp.), imperiled G2 (21 spp.), and vulnerable G3 (21 spp.). Five species were ranked GH (possibly extinct), and eight were designated GNR or GU (unranked). One species, *Oxyethira simulatrix*, is not found currently in NatureServe (2024) and should be added to the system, as it was reported as a new United States country record by Harris et al. (2012), based on specimens collected from numerous localities on the Florida peninsula. Comparatively few of the Florida caddisfly species (65 spp.) have been given state conservation status rankings. These rankings, assigned by the Florida Natural Areas Inventory (FNAI), focus on those species considered to be potentially threatened or endangered. The state rankings for Florida include 27 species that are considered critically imperiled (S1), 24 ranked as imperiled (S2), and seven which have been deemed vulnerable (S3). Two species are ranked as apparently secure (S4).

Four of five species globally ranked GH (possibly extinct) are ranked as possibly extirpated (SH) by FNAI. The conservation status rankings of these four GH/SH species, comprising three hydroptilid species (*Ochrotrichia okaloosa*, *O. provosti*, *Oxyethira kingi*) and one leptocerid (*Oecetis pratelia*), were reviewed by Rasmussen et al. (2008a). One species ranked GH, the microcaddisfly *Ochrotrichia apalachicola*, is ranked SX (presumed extirpated). The species was reported by Harris et al. (2012) from several spring-fed headwater streams in the western panhandle of Florida; we recommend that its rankings be changed to G3/S3 (vulnerable).

Of the 62 Florida species that are currently considered globally vulnerable or imperiled (G1, G2, G3), many are narrow-range endemics (including 18 species endemic to Florida) and restricted to geographically isolated habitats such as oligotrophic sandhill lakes, steephead ravine streams (Rasmussen, 2004), and larger calcareous spring-fed systems such as the Chipola River (Denson et al. 2016). As part of the review process for species petitioned for federal listing, Rasmussen and Harris (2016) provided detailed information and assessments for five G1 or G2 species: *Agarodes logani* (Sericostomatidae), *Hydroptila sykorai* (Hydroptilidae), *Lepidostoma morsei* (Lepidostomatidae), *Oxyethira setosa* (Hydroptilidae), and *Triaenodes tridonta* (Leptoceridae).

Lastly, we recommend that the five new species described in this publication be assigned global/state conservation rankings as follows: *Beraea jennyae* sp. nov. (G1/S1), *Protoptila chipolensis* sp. nov. (G3/S3), *Hydroptila aviforma* sp. nov. (G3/S3), *Ceraclea pescadori* sp. nov. (G3/S3), and *Oecetis densoni* sp. nov. (G3/S3).

## ﻿Conclusions

Based on published distributional records dating back to the mid-19^th^ century and studies on caddisfly biodiversity in Florida that we have conducted over the last 30 years, we provide an updated and accurate characterization of Florida’s Trichoptera fauna. The annotated checklist presented includes county records for 220 species within 46 genera and 19 families. Seven species new to science are documented, with original descriptions provided for five of them. Thirty-four of 220 species (15%) are thought to be endemic to the state. As such, the caddisfly fauna of Florida represents a diverse and unique component of the caddisfly fauna of the Southeastern Coastal Plain within the eastern Nearctic region. Despite the close proximity of Florida to Caribbean islands (e.g., Cuba) of the Neotropics, the composition of the Florida fauna shows little similarity to insular island faunas due to geographic isolation from Neotropical freshwater habitats.

Geographic distributions of caddisfly species at the county level indicated that the panhandle region of Florida (counties 1–18) contains a highly diverse fauna (213 spp.), including 23 species endemic to Florida’s panhandle. By comparison, the Florida peninsula (counties 19–67) contains far fewer species (131 spp.) and fewer species endemic to the peninsula (5 spp.). Caddisfly diversity on the peninsula was found to be much lower in the southern counties than in counties within the northern half of the peninsula. The large differences in the alpha diversity of these regions is likely determined by a combination of factors involving regional differences in the diversity of aquatic habitats, north/south temperature gradient, and the influence of historical biogeography that have led to confinement of cool-adapted taxa within northern Florida, along with the presence of a strictly warm-adapted fauna in south Florida.

The continued discovery of species new to science, as well as new state and county records, are indicators that our understanding of the caddisfly fauna of Florida is far from complete. Future advancement in understanding the diversity and distribution of Florida caddisflies is best achieved by targeting counties that have been under-collected and also by sampling unique habitats statewide throughout different seasons using traditional caddisfly collecting methods such as light trapping, along with less commonly used collecting methods such as malaise traps to document additional species that may not be susceptible to capture using lights. Employing mitochondrial DNA barcoding methods will likewise expedite species discovery by identifying genetically distinct taxa, including otherwise cryptic species. In addition, DNA barcoding offers the ability to rapidly and accurately associate life stages and sexes, permitting their descriptions (Orfinger et al. 2022). Integration of resulting sequence data into public databases such as the Barcode of Life Database strengthens the existing barcoding library available for others to leverage molecular identification methods in Florida Trichopterology and beyond.

Fortunately, many areas of Florida where we have documented high caddisfly biodiversity are under management and protection to various degrees by state (e.g., state forests, water management district lands, state parks) and national (e.g., national forests, military bases) governmental entities. By including information in this publication on the conservation status of Florida species, we are advocating for increased partnering efforts between conservation organizations (e.g., NatureServe, Florida Natural Areas Inventory), governmental agencies (e.g., U.S. Fish and Wildlife Service, Florida Fish and Wildlife Conservation Commission), and taxonomic specialists to better protect and conserve aquatic resources and caddisfly biodiversity within the state. It is our hope that the next generation of aquatic scientists will find this baseline information useful as a foundation to develop future studies of caddisfly biodiversity, especially as Florida’s caddisfly biodiversity comes under increasing threats due to climate change and degradation of freshwater habitats.

## Supplementary Material

XML Treatment for Protoptila chipolensis

XML Treatment for Hydroptila aviforma

XML Treatment for Beraea jennyae

XML Treatment for Ceraclea pescadori

XML Treatment for Oecetis densoni

## ﻿Additional information

### Author ORCIDs

Andrew K. Rasmussen https://orcid.org/0009-0005-9549-289X

Dana R. Denson https://orcid.org/0000-0003-0401-789X

Alexander B. Orfinger https://orcid.org/0000-0002-4907-3150

Steven C. Harris https://orcid.org/0000-0002-6432-7462

### Data availability

All of the data that support the findings of this study are available in the main text or Supplementary Information.

## Supplementary materials

Supplementary material 1 Checklist of Florida Trichoptera: presence by county matrix Data type xlsx https://binary.pensoft.net/file/1488199This dataset is made available under the Open Database License (http://opendatacommons.org/licenses/odbl/1.0/). The Open Database License (ODbL) is a license agreement intended to allow users to freely share, modify, and use this Dataset while maintaining this same freedom for others, provided that the original source and author(s) are credited.Andrew K. Rasmussen, Dana R. Denson, Alexander B. Orfinger, Steven C. Harris

Supplementary material 2 Pairwise genetic divergence (p-distance) between *Oecetis densoni* sp. nov. and *O. cinerascens* (Hagen) Data type docx https://binary.pensoft.net/file/1488200This dataset is made available under the Open Database License (http://opendatacommons.org/licenses/odbl/1.0/). The Open Database License (ODbL) is a license agreement intended to allow users to freely share, modify, and use this Dataset while maintaining this same freedom for others, provided that the original source and author(s) are credited.Andrew K. Rasmussen, Dana R. Denson, Alexander B. Orfinger, Steven C. Harris

Supplementary material 3 Neighbor Joining tree for mtCOI barcoding sequence data of *Oecetis* species examined during the course of this study Data type tif Explanation note Scale bar indicates genetic distance. https://binary.pensoft.net/file/1488201This dataset is made available under the Open Database License (http://opendatacommons.org/licenses/odbl/1.0/). The Open Database License (ODbL) is a license agreement intended to allow users to freely share, modify, and use this Dataset while maintaining this same freedom for others, provided that the original source and author(s) are credited.Andrew K. Rasmussen, Dana R. Denson, Alexander B. Orfinger, Steven C. Harris
