# Supplementary material for: ﻿Diversity and distribution of the Trichoptera of Florida, United States, with descriptions of five new species
Source: Zookeys. 2025 Dec 10;1263:389–439. doi: 10.3897/zookeys.1263.147317 (PMC12712626; doi:10.3897/zookeys.1263.147317)
Supplement: Supplementary material 2 — Pairwise genetic divergence (p-distance) between Oecetis densoni sp. nov. and O. cinerascens (Hagen) [file zookeys-1263-389_article-147317__-s002.docx]

**Supplementary Table 2.** Pairwise genetic divergence (p-distance) between *Oecetis densoni* sp. nov. and *O. cinerascens* (Hagen).

|  | FLCAD148-09\|*O. cinerascens* | PKCAD021-07\|*O. cinerascens* | FLCAD130-09\|*O. cinerascens* | FLCAD056-08\|*O. cinerascens* | FLCAD120-09\|*O. densoni* sp. nov. |
| --- | --- | --- | --- | --- | --- |
| FLCAD148-09\|*O. cinerascens* |  |  |  |  |  |
| PKCAD021-07\|*O. cinerascens* | 0.003976 |  |  |  |  |
| FLCAD130-09\|*O. cinerascens* | 0.001579 | 0.003979 |  |  |  |
| FLCAD056-08\|*O. cinerascens* | 0.002372 | 0.004782 | 0.002374 |  |  |
| FLCAD120-09\|*O. densoni* sp. nov. | 0.066837 | 0.069062 | 0.064805 | 0.065795 |  |
| FLCAD140-09\|*O. densoni* sp. nov | 0.065844 | 0.068062 | 0.063816 | 0.064805 | 0.000788 |
