# Supplementary figures and images for: ﻿Diversity and distribution of the Trichoptera of Florida, United States, with descriptions of five new species
Source: Zookeys. 2025 Dec 10;1263:389–439. doi: 10.3897/zookeys.1263.147317 (PMC12712626; doi:10.3897/zookeys.1263.147317)

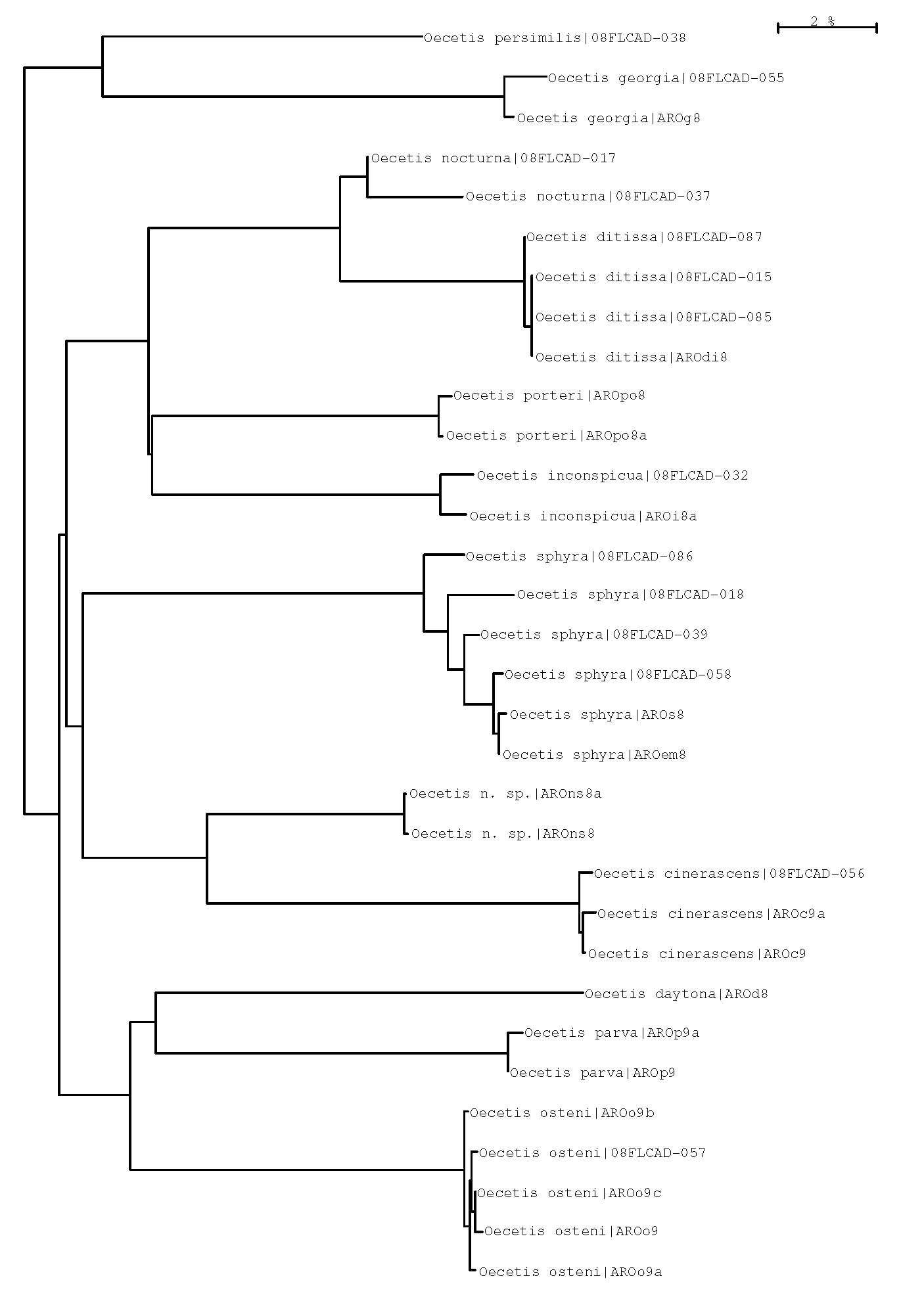

Supplement: Supplementary material 3 — Neighbor Joining tree for mtCOI barcoding sequence data of Oecetis species examined during the course of this study [file zookeys-1263-389_article-147317__-s003.tif]
